# PD-1 blockade enhances chemotherapy toxicity in oesophageal adenocarcinoma

**DOI:** 10.1038/s41598-022-07228-x

**Published:** 2022-02-28

**Authors:** Maria Davern, Rebecca M. O’ Brien, Jason McGrath, Noel E. Donlon, Ashanty M. Melo, Croí E. Buckley, Andrew D. Sheppard, John V. Reynolds, Niamh Lynam-Lennon, Stephen G. Maher, Joanne Lysaght

**Affiliations:** 1 Cancer Immunology and Immunotherapy Group, Department of Surgery, Trinity Translational Medicine Institute and Trinity St. James’s Cancer Institute, St. James’s Hospital, Trinity College Dublin, Dublin 8, Ireland; 2 Translational Radiobiology and Diagnostics Group, Department of Surgery, Trinity Translational Medicine Institute, St. James’s Hospital, Dublin 8, Ireland; 3 Department of Surgery, Trinity Translational Medicine Institute, St. James’s Hospital, Trinity College Dublin, Dublin 8, Ireland; 4 Cancer Chemoradiation Group, Department of Surgery, Trinity Translational Medicine Institute, St. James’s Hospital, Dublin 8, Ireland

**Keywords:** Cancer immunotherapy, Chemotherapy

## Abstract

Chemotherapy upregulates immune checkpoint (IC) expression on the surface of tumour cells and IC-intrinsic signalling confers a survival advantage against chemotherapy in several cancer-types including oesophageal adenocarcinoma (OAC). However, the signalling pathways mediating chemotherapy-induced IC upregulation and the mechanisms employed by ICs to protect OAC cells against chemotherapy remain unknown. Longitudinal profiling revealed that FLOT-induced IC upregulation on OE33 OAC cells was sustained for up to 3 weeks post-treatment, returning to baseline upon complete tumour cell recovery. Pro-survival MEK signalling mediated FLOT-induced upregulation of PD-L1, TIM-3, LAG-3 and A2aR on OAC cells promoting a more immune-resistant phenotype. Single agent PD-1, PD-L1 and A2aR blockade decreased OAC cell viability, proliferation and mediated apoptosis. Mechanistic insights demonstrated that blockade of the PD-1 axis decreased stem-like marker ALDH and expression of DNA repair genes. Importantly, combining single agent PD-1, PD-L1 and A2aR blockade with FLOT enhanced cytotoxicity in OAC cells. These findings reveal novel mechanistic insights into the immune-independent functions of IC-intrinsic signalling in OAC cells with important clinical implications for boosting the efficacy of the first-line FLOT chemotherapy regimen in OAC in combination with ICB, to not only boost anti-tumour immunity but also to suppress IC-mediated promotion of key hallmarks of cancer that drive tumour progression.

## Introduction

Oesophageal adenocarcinoma (OAC) is the predominant subtype of oesophageal cancer in Western countries^1^. Moreover, OAC is an exemplar model of an obesity-driven cancer and as such its incidence is rapidly increasing in parallel with the rising level of obesity^2^. Response rates to the standard of care FLOT (5-fluorouracil (5-FU), oxaliplatin and docetaxel) chemotherapy regimen remain low with a complete pathologic response rate of 16.6%^3^ and subsequent clinical outcomes are dismal with a median overall survival rate of 50 months^3^ and 5-year overall survival rates as low as 15–40% depending on tumour stage at clinical presentation^4^. Improvements in the efficacy of first-line chemotherapy regimens were achieved through combining immune checkpoint blockade (ICB) with chemotherapy as depicted in the recent findings from the phase III Checkmate 649 trial, which demonstrated the synergy between nivolumab and first-line chemotherapy (FOLFOX and XELOX) in previously untreated oesophagogastric junctional adenocarcinoma (OGJ) patients (n = 1581), in which a significant improvement in overall survival in patients with a PD-L1 combined positive score of 5 or greater was observed (14.4 months (nivolumab plus chemotherapy arm) vs. 11.1 months (chemotherapy arm))^5^. Furthermore, the nivolumab plus chemotherapy arm also reduced the risk of death by 29% (HR, 0.71; 98.4% CI, 0.59–0.86; *p* < 0.0001)^5^. The findings from this trial highlight the potential synergy that can be exploited between chemotherapy and ICB in the first-line setting. Rational explanations for the improvement in overall survival are likely attributed to the ICB-mediated reinvigoration of anti-tumour immune responses. Moreover, mounting evidence in the literature suggest that the FOLFOX/XELOX chemotherapy regimens^6–11^ may possess immunostimulatory properties with the potential ability to convert a ‘cold non-inflamed’ into a ‘hot inflamed’ tumour microenvironment. 5-FU and oxaliplatin chemotherapies which comprise the FLOT regimen, have been shown to induce immunogenic tumour cell death in several cancer types^6–10^. Therefore, addition of ICB to the FLOT/XELOX regimen likely reinvigorated exhausted anti-tumour immunity and prevented exhaustion of chemotherapy-induced anti-tumour immune responses, producing synergistic amplification of anti-tumour immunity, which translated into durable clinical responses in gastroesophageal cancer patients. Oxaliplatin induced immunogenic cell death in colorectal cancer^6^ and lung carcinoma^7,8^, and 5-FU induced immunogenic cell death in colon carcinoma^9^ and gastric cancer^10^. Immunogenic cell death is a particular modality of cell death that can be triggered by selected anticancer chemotherapeutics^11^. Tumour cells undergoing immunogenic cell death are characterised by the release or exposure of damage-associated molecular patterns (DAMPs) that stimulate the attraction, activation and maturation of dendritic cells and eventually the antigen-specific priming of cytotoxic T lymphocytes^12^. This can induce an adaptive anticancer immune response that targets residual cancer cells with the same antigenic profile^13^.

Aside from ICB-mediated inhibition of immune evasion, another possible explanation of the beneficial outcomes with ICB may be attributed to their effect on immune-independent hallmarks of cancer. Abundant evidence in the literature has highlighted that use of ICB to inhibit PD-L1^14^, A2aR^15^ or TIM-3^16^ tumour cell-intrinsic signalling can suppress tumour cell invasion and migration and subsequent metastasis in a range of cancer models of gastric cancer, lung cancer and cervical cancer. Moreover, the PD-1 axis has been implicated in conferring chemo(radio)-resistance through promotion of stem-like characteristic in lung cancer^17^ and enhancement of radiation-induced DNA repair in osteosarcoma^18^. Additionally, PD-L1 signalling induced proliferation of tumour cells in a range of cancer types including hepatocellular carcinoma^19^, melanoma and ovarian cancer^20^.

However, the mechanistic role of PD-1, PD-L1 and A2aR tumour cell-intrinsic signalling in the context of OAC remains to be investigated. Therefore, this study explores how the first-line FLOT chemotherapy regimen alters IC expression profiles of OAC cells longitudinally. FLOT comprises of a unique cocktail of chemotherapies including 5-FU (an anti-metabolite), oxaliplatin (DNA intercalator) and docetaxel (taxane) with specific mechanisms of action that target distinct phases of the cell cycle. 5-FU exerts its anticancer effects through inhibition of thymidylate synthase and incorporation of its metabolites into RNA and DNA resulting in damage, generating considerable amounts of cellular genotoxic stress and hindering the normal functioning and homeostasis of cellular processes that require these RNA and DNA^21^. Oxaliplatin, a platinum-based chemotherapy intercalates with cellular DNA forming platinum–DNA adducts, which induce DNA damage and block DNA replication^22^. Docetaxel inhibits microtubular depolymerization, and attenuated the effects of bcl-2 and bcl-xL gene expression which ultimately culminates in G2/M phase cell cycle arrest apoptotic cell death^23^.

The effect of single agent ICB on the viability of OAC cells is assessed including the ability of ICB to enhance FLOT chemotherapy cytotoxicity in OAC cells. Mechanistic insights are also provided surrounding the regulation of FLOT-induced upregulation of ICs on OAC cells. This study profiles the effect of inhibiting oncogenic signalling pathways such as the mitogen-activated extracellular signal-regulated kinase (MEK) and STAT3 which are frequently activated in cancer on the expression profile of ICs in OAC cells^24,25^. The MEK pathway^24^ and the IL-6/JAK/STAT3 pathway^25^ are triggered by either growth factors or activating mutations of major oncogenic proteins in this pathway, the most common being Ras and Raf for MEK signalling. Both pathways are aberrantly hyperactivated in many types of cancer driving proliferation, survival, invasiveness, and metastasis of tumour cells, while strongly suppressing the antitumour immune response^24,25^. A plethora of studies has identified a role for MEK signalling in tumour cells as a mechanism for conferring chemoresistance, whereby attenuation of MEK signalling overcame chemoresistance in tumour types such as nasopharyngeal carcinoma, oral squamous cell carcinoma^26,27^ and breast cancer^28^. Furthermore, MEK signalling has also been shown to regulate PD-L1 expression in tumour cells specifically via epidermal growth factor- and interferon-gamma-induced PD-L1 expression in lung adenocarcinoma cells and blockade of MEK1/2 attenuated PD-L1 upregulation^29^.

A deeper understanding of the immune-independent effects of IC signalling in OAC cells is also assessed including effects on tumour cell proliferation, DNA repair and expression of cancer stem-like properties with and without FLOT treatment. Collectively, the findings of this study will offer important insights for the design of synergistic ICB-chemotherapy combinations for OAC patients.

## Methods

### Culture of cell lines

OE33 and SK-GT-4 oesophageal adenocarcinoma cells were purchased from European Collection of Cell Cultures. OE33 cells and SK-GT-4 cells were grown in RPMI 1640 medium with 2 mM L-glutamine (ThermoFisher Scientific, Ireland) and supplemented with 1% (v/v) penicillin–streptomycin (50 U/ml penicillin 100 μg/ml streptomycin (P/S)) and 10% (v/v) foetal bovine serum (FBS) (ThermoFisher Scientific, Ireland) and detached using trypsin–EDTA solution (Gibco, ThermoFisher Scientific, Ireland). All cell lines were maintained in a humidified chamber at 37 °C 5% CO_2_ and were tested regularly to ensure mycoplasma negativity. The OE33 cell line was established from a poorly differentiated stage IIA adenocarcinoma of the lower oesophagus (Barrett’s metaplasia) of a 73-year old female patient. Whereas, the SK-GT-4 cell line originated from a well differentiated oesophageal adenocarcinoma arising in Barrett’s epithelium from an 89-year-old Caucasian male. Timme et al., demonstrated that STAT3 signalling is weakly constitutively activated in OE33 cells and is more preferentially activated in oesophageal squamous cell carcinomas. Nonetheless, STAT3 blockade or knockout has been shown to inhibit OE33 tumour cell survival, proliferation, migration, and increase apoptosis^30,31^. Mutations in the KRAS gene have been identified in the pre-malignant condition Barrett’s oesophagus and in OAC lesions^32^. The MAPK pathway mutational status in the OE33 and SK-GT-4 cell lines are limited. Although there are no reported mutations in the MAPK pathway in the OE33 cell line it has been identified that the OE33 cells have elevated gene copy numbers of Aurora-A^33^ which has been identified as an enhancer of MAPK signalling^34^. Additionally, a missense mutation in the KRAS gene has also been found in the SK-GT-4 cell line according to the canSAR Black cell line database, which details known mutations in cell lines however, the functional consequences have not been elucidated.

### Flow cytometry staining

OE33 cells and SK-GT-4 cells were treated with nivolumab (10 μg/ml, Opdivo, Bristol-Myers Squibb, USA), atezolizumab (10 μg/ml, Tecentriq, Roche, USA), A2aR antagonist (3 μM, ZM 241385 catalogue # 1036/10 Bio-techne, USA) or a MEK inhibitor (0.001 μM, U0126 Merck, USA) in the absence or presence of FLOT chemotherapy regimen. Doses of the FLOT chemotherapy regimen were pre-optimised to reduce the viability of cells by 50% (IC_50_ dose) as previously described (OE33 cells: 0.8249 µM 5-FU, 2 µM oxaliplatin and 0.001 µM of docetaxel and SK-GT-4 cells: 50 µM 5-FU, 10 µM oxaliplatin and 0.01 µM docetaxel) or cells were treated with the vehicle control (0.002% DMSO, 0.2% H_2_O)^35^. Trypsinised OE33 cells or SK-GT-4 cells were stained with Zombie Aqua™, Violet™ or NIR™ viability dye (BioLegend, USA). Antibodies used for staining included LAG-3-FITC, CD160-PerCPCy5.5, PD-1-PE/Cy7, TIGIT-PE/Cy7 (BioLegend, USA), TIM-3-AF647, PD-L1-FITC, PD-L2-PE (BD Bioscience, USA) and A2aR-PE (Bio-techne, USA). OAC cells were resuspended in FACS buffer and acquired using BD FACS CANTO II (BD Biosciences, USA) using Diva software and analysed using FlowJo v10 software (TreeStar Inc.).

### Detection of Ki67 staining by flow cytometry

Cells were collected by trypsinizatfion, fixed in ice-cold 70% ethanol (Merck, Darmstadt, Germany) and incubated at room temperature for 30 min. The fixative was decanted after centrifugation at 1,300 RPM for 3 min and cells were resuspended in 100 μl of FACS buffer and stained with 1 μl of Ki67-AF647 (BioLegend, USA). OAC cells were washed and resuspended in FACS buffer and acquired using BD FACS CANTO II (BD Biosciences, USA) using Diva software and analysed using FlowJo v10 software (TreeStar Inc.).

### Cell viability CCK-8 assay

A CCK-8 assay (Sigma, USA) was used to assess the effect of ICB with and without FLOT chemotherapy regimen on the proliferation rate of OE33 cells and SK-GT-4 cells. 5 × 10^3^ OAC cells were adhered in a 96 well plate at 37 °C, 5% CO2 overnight. The media was removed, and cells were cultured for 48 h in complete RPMI in the absence or presence of nivolumab (10 μg/ml, Opdivo, Bristol-Myers Squibb, USA), atezolizumab (10 μg/ml, Tecentriq, Roche, USA), A2aR antagonist (3 μM, Bio-techne, USA) with and without the FLOT chemotherapy regimen (IC_50_ dose). 5 μl of CCK-8 solution was added to each well, followed by a 1.5 h incubation in the dark at 37 °C, 5% CO2. The optical density at 450 nm and 650 nm (reference wavelength) was measured using the Versa Max microplate reader (Molecular Devices, Sunnyvale, CA, USA) to determine a viable cell number. All of the data were analysed from three independent experiments.

### Annexin V and propidium iodide assay

Apoptosis and necrosis was measured using annexin V (AV)-FITC and propidium iodide (PI, (Invitrogen, Carlsbad, CA, USA), staining and was assessed by flow cytometry. OE33 cells and SK-GT-4 cells were cultured for 48 h in complete RPMI in the absence or presence of nivolumab (10 μg/ml, Opdivo, Bristol-Myers Squibb, USA), atezolizumab (10 μg/ml, Tecentriq, Roche, USA), A2aR antagonist (3 μM, Bio-techne, USA) with and without the FLOT chemotherapy regimen (IC_50_ dose). Cells were stained with Annexin V-FITC (BioLegend, USA) and 1:4000 PI (Invitrogen, Carlsbad, CA, USA), and samples were acquired using BD FACS CANTO II (BD Biosciences, USA) using Diva software and analysed using FlowJo v10 software (TreeStar, Inc., Ashland, Oregon).

### Detection of γH2AX by flow cytometry

After treatment, cells were collected by trypsinization, fixed in ice-cold 70% ethanol (Merck, Darmstadt, Germany), and incubated at room temperature for 30 min. The fixative was decanted after centrifugation at 1,300 RPM for 3 min with 2 ml of PBS containing 2% FBS. Cells were resuspended in 100 μl γH2AX staining (1:100 dilution of antibody, BioLegend, USA) solution [Triton X100 (0.1%), FBS (2%)] for 2 h at room temperature. OAC cells were resuspended in FACS buffer and acquired using BD FACS CANTO II (BD Biosciences, USA) using Diva software and analysed using FlowJo v10 software (TreeStar Inc.).

### RNA isolation and quantification

Cells were seeded at a density of 3 × 10^6^ cells in a T75 flask in 11 ml of cRPMI and allowed to adhere overnight. Following drug treatment RNA was isolated from cell lines using the TRI Reagent® method. The RNA pellet was re-suspended in 30 μl RNAase free molecular grade H_2_O and stored at − 80 °C. RNA quantification was determined spectrophotometrically, using a Nanodrop 1000 spectrophotometer (version 3.1.0, Nanodrop technologies, DE, USA).

### cDNA synthesis

For cell line samples, total RNA (1 μg total RNA) was reverse transcribed to cDNA using the manufacturer’s instructions for the cDNA Reverse Transcription Kit from eBioscience USA (4368814). In brief, to anneal the primers to the RNA, a master mix containing RNaseOUT 25X dNTP Mix 10 mm, (10 mM, prepared as a 1:1:1:1 ratio of dATP, dGTP, dTTP and dCTP), 10 × rt random primers, Bioscript reverse transcriptase (200units/μl) and 10X Bioscript Reaction Buffer in RNase-free water was added to each sample and the samples were incubated for 10 min at 25 °C, for 120 min at 37 °C then for 5 min at 85 °C and held at 4 °C. The resulting cDNA was stored at − 20 °C.

### Quantitative real time PCR and analysis

qPCR was performed using TaqMan primer probes (*MLH1, SMUG1, PARP1, MMS19* (Roche, USA)) and a Quant Studio 5 real-time thermal cycler (Thermo Fisher Scientific, Ireland). 18S (eBioscience, USA) was used as an endogenous control for data normalization. Data analysis was performed using Thermofisher Scientific Connect qPCR application software.

### Aldehyde dehydrogenase (ALDH) assay

Aldehyde dehydrogenase (ALDH) enzyme activity was assessed using the Aldefluor® assay (Stem Cell Technologies, USA), according to the manufacturer’s instructions. Briefly, cells were trypsinised and resuspended at a density of 1 × 10^6^ cells/mL in Aldefluor® assay buffer containing ALDH substrate (bodipy-aminoacetaldehyde) (5 μL/mL). Immediately following this, half of the resuspended cells were added to a tube containing the ALDH inhibitor diethylaminobenzaldehyde (DEAB) to provide a negative control. Cells were acquired using BD FACS CANTO II (BD Biosciences) using Diva software and analysed using FlowJo v10 software (TreeStar Inc.).

### Statistical analysis

Data were analysed using GraphPad Prism version 9 (GraphPad Prism, San Diego, CA, USA) and was expressed as mean ± SEM. A two-way Anova was used to compare between multiple groups across longitudinal timepoints using Benjamini and Hochberg post-hoc tests to correct for false discovery rate. Kruskall-Wallis was used to compare between 2 groups. Statistical significance was determined as *p* ≤ 0.05.

## Results

### Chemotherapy-induced upregulation of specific ICs on the surface of OAC cells

**Figure 1:**
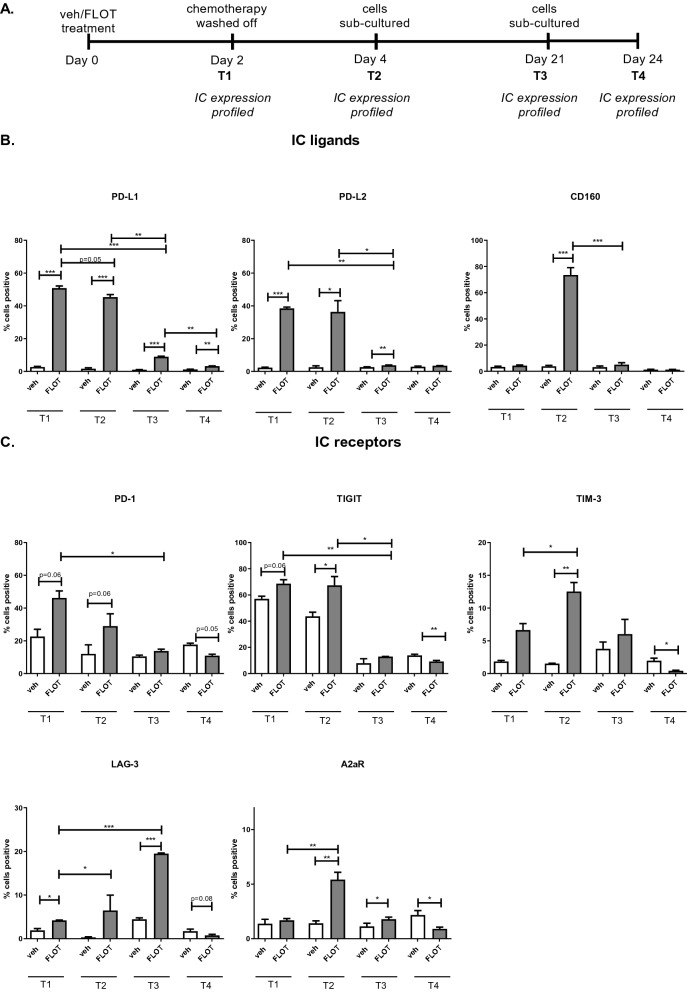
FLOT dynamically upregulates ICs on the surface of OAC cells longitudinally. OE33 cells were treated with vehicle control (veh) or FLOT for 48 h (T1), washed twice and allowed to grow for an additional 48 h (T2) after which the cells were sub-cultured in new flasks and left to recover for 3 weeks (T3). Following complete recovery, the cells were sub-cultured 1 in 2 and screened for IC expression 3 days later (T4). IC ligand and receptor expression was profiled on the surface of OE33 cells longitudinally by flow cytometry at T1, T2, T3 and T4. (**A**) Represents a schematic of experimental setup. (**B**) IC ligands expression profiles for PD-L1, PD-L2 and CD160. (**C**) IC receptor profiles for PD-1, TIGIT, TIM-3, LAG-3 and A2aR. Experiments conducted for an n = 4 independent experimental repeats using singlet technical replicates. Two-way Anova using Benjamini and Hochberg to correct for false discovery rate, *p < 0.05, **p < 0.01 and ***p < 0.001.

We have previously demonstrated that first-line chemotherapy combinations FLOT and CROSS (carboplatin and paclitaxel) chemotherapies upregulated IC ligands and receptors on the surface of OAC cells following 48 h treatment *in vitro*^35^. Therefore, we sought to investigate how long this chemotherapy-induced upregulation of ICs on the surface of OAC cells is maintained by longitudinally profiling IC expression on the surface of OE33 cells following 48 h treatment with vehicle control or FLOT chemotherapy regimen (Fig. 1A). The well-known PD-L1 and PD-L2 IC ligands were included in this screen as well as a more novel IC ligand CD160 which is expressed on T cells and NK cells and possesses two binding partners MHC I or HVEM. CD160 promotes NK cell cytotoxicity and IFN-γ production, but the function of CD160 on CD8^+^ T cells remains unclear with certain studies supporting a coinhibitory role^36^ and others a costimulatory role^37^. A recent study published by our group^35^ also identified that CD160 IC ligand is expressed on tumour cells in OAC patients and in OAC tumour cell lines. Therefore, we sought to further interrogate the effect of chemotherapy treatment on the longitudinal expression profile of IC ligands.

48 h treatment with FLOT significantly increased PD-L1 expression on the surface of OE33 cells compared with the vehicle control at 48 h (50.85 ± 1.2 vs. 2.65 ± 0.3%, *p* < 0.0001, Fig. 1B). FLOT-induced PD-L1 upregulation on the surface of OE33 cells remained upregulated at 4 days (45.35 ± 1.5 vs. 1.63 ± 0.5%, *p* < 0.0001) and 21 days (8.88 ± 0.3 vs. 0.97 ± 0.1%, *p* < 0.0001, Fig. 1B) post-treatment compared with the vehicle control (Fig. 1B). Following subculture of recovered FLOT-treated OE33 cells PD-L1 was significantly upregulated compared with the vehicle control (3.15 ± 0.2 vs. 1.15 ± 0.1, *p* = 0.007, Fig. 1B).

Comparably, 48 h treatment with FLOT significantly increased PD-L2 expression on the surface of OE33 cells compared with the vehicle control at 48 h (38.45 ± 0.7 vs. 2.28 ± 0.2%, *p* < 0.0001, Fig. 1B). FLOT-induced PD-L2 upregulation on the surface of OE33 cells remained upregulated at 4 days (36.33 ± 6.8 vs. 2.5 ± 0.9, *p* = 0.01) and 21 days (3.7 ± 0.1 vs. 2.58 ± 0.2, *p* = 0.001, Fig. 1B) post-treatment compared with the vehicle control (Fig. 1B). In contrast to PD-L1, following subculture of recovered (day 24) FLOT-treated OE33 cells PD-L2 expression had returned to baseline with no significant difference when compared with the vehicle control (Fig. 1B).

Although 48 h treatment with FLOT did not significantly alter CD160 expression on the surface of OE33 cells compared with the vehicle control at 48 h, CD160 was significantly upregulated 4 days post-FLOT treatment compared with the vehicle control (73.53 ± 5.6 vs. 3.75 ± 0.7, *p* = 0.0008, Fig. 1B). However, CD160 expression had returned to baseline 21 days post-FLOT treatment and following subculture of recovered FLOT-treated OE33 cells with no significant difference when compared with the vehicle control (Fig. 1B).

The effect of chemotherapy treatment on the longitudinal expression profile of an array of well-known IC receptors were also assessed in this screen and included PD-1, TIGIT, TIM-3, LAG-3 and A2aR. Following 48 h treatment with FLOT, PD-1 expression increased on the surface of OE33 cells on day 2 (46.18 ± 4.3 vs. 22.60 ± 4.4, *p* = 0.06) and on day 4 compared with the vehicle control (28.95 ± 7.5 vs. 12.02 ± 5.5%, *p* = 0.06, Fig. 1C). Furthermore, following subculture of recovered FLOT-treated OE33 cells there was a reduction in PD-1 expression compared with the vehicle control (10.92 ± 0.8 vs. 17.63 ± 0.8, *p* = 0.06, Fig. 1C).

TIGIT expression increased on the surface of OE33 cells compared with the vehicle control at 48 h (68.58 ± 3.0 vs. 57.0 ± 2.0%, *p* = 0.06, Fig. 1C) and remained upregulated at 4 days (67.28 ± 6.8 vs. 43.65 ± 3.1, *p* = 0.01). However, 21 days post-FLOT treatment TIGIT expression returned to baseline and was comparable with the vehicle control (Fig. 1C). Following subculture of recovered FLOT-treated OE33 cells, TIGIT was significantly decreased compared with the vehicle control (9.17 ± 0.8 vs. 13.80 ± 0.9%, *p* = 0.004, Fig. 1C).

Similar to TIGIT, following 48 h treatment with FLOT, TIM-3 expression increased on the surface of OE33 cells compared with the vehicle control at 48 h (6.65 ± 0.9 vs. 1.8 ± 0.1, *p* = 0.09, Fig. 1C) and remained upregulated at 4 days (12.53 ± 1.3 vs. 1.51 ± 0.1%, *p* = 0.003) (Fig. 1C). Likewise, to TIGIT, following subculture of recovered FLOT-treated OE33 cells was a significant reduction in TIM-3 expression compared with the vehicle control (0.41 ± 0.1 vs. 1.95 ± 0.4%, *p* = 0.05, Fig. 1C).

Following 48 h treatment with FLOT, LAG-3 was significantly increased on the surface of OE33 cells compared with the vehicle control at 48 h (4.19 ± 0.1 vs. 1.90 ± 0.4%, *p* = 0.03, Fig. 1C) and remained upregulated at 21 days (19.50 ± 0.1 vs. 4.43 ± 0.3%, *p* = 0.009, Fig. 1C). Similar, to findings observed for TIGIT and TIM-3, following subculture of recovered FLOT-treated OE33 cells, LAG-3 expression decreased compared with the vehicle control (0.76 ± 0.2 vs. 1.71 ± 0.4%, *p* = 0.06, Fig. 1C).

Although 48 h treatment with FLOT did not significantly alter A2aR expression on the surface of OE33 cells compared with the vehicle control at 48 h, A2aR was significantly upregulated 4 days post-FLOT treatment (5.41 ± 0.6 vs. 1.41 ± 0.2%, *p* = 0.004) and 21 days (1.78 ± 0.1 vs. 1.12 ± 0.2%, *p* = 0.02, Fig. 1C). Similarly, following subculture of recovered FLOT-treated OE33 cells, A2aR expression was decreased compared with the vehicle control (0.90 ± 0.1 vs. 2.16 ± 0.4%, *p* = 0.06, Fig. 1C). Co-expression analysis of multiple inhibitory ICs was also assessed longitudinally and depicted similar trends (Figure S1).

In summary, several ICs were significantly upregulated on the surface of OE33 cells longitudinally including PD-L1, PD-L2, CD160, TIGIT, TIM-3, LAG-3 and A2aR. In addition, PD-L1 remained increased compared with the vehicle control following subculture of FLOT-recovered OE33 cells at 24 days, whilst PD-L2 and CD160 returned to baseline expression levels. PD-1, TIGIT, TIM-3, LAG-3 and A2aR expression were decreased compared with the vehicle control following subculture of FLOT-recovered OE33 cells at 24 days.

### Pro-survival MEK signalling upregulates ICs on the surface of OAC cells following chemotherapy treatment

Chemotherapy-induced upregulation of ICs on the surface of OAC cells suggests these tumour cells may be employing ICs as an adaptive survival mechanism to overcome genotoxic stress. However, the signalling pathways mediating FLOT-induced IC upregulation remain unknown. Therefore, we sought to investigate if the pro-survival signalling pathway MEK may be regulating the chemotherapy-induced upregulation of ICs.

**Figure 2:**
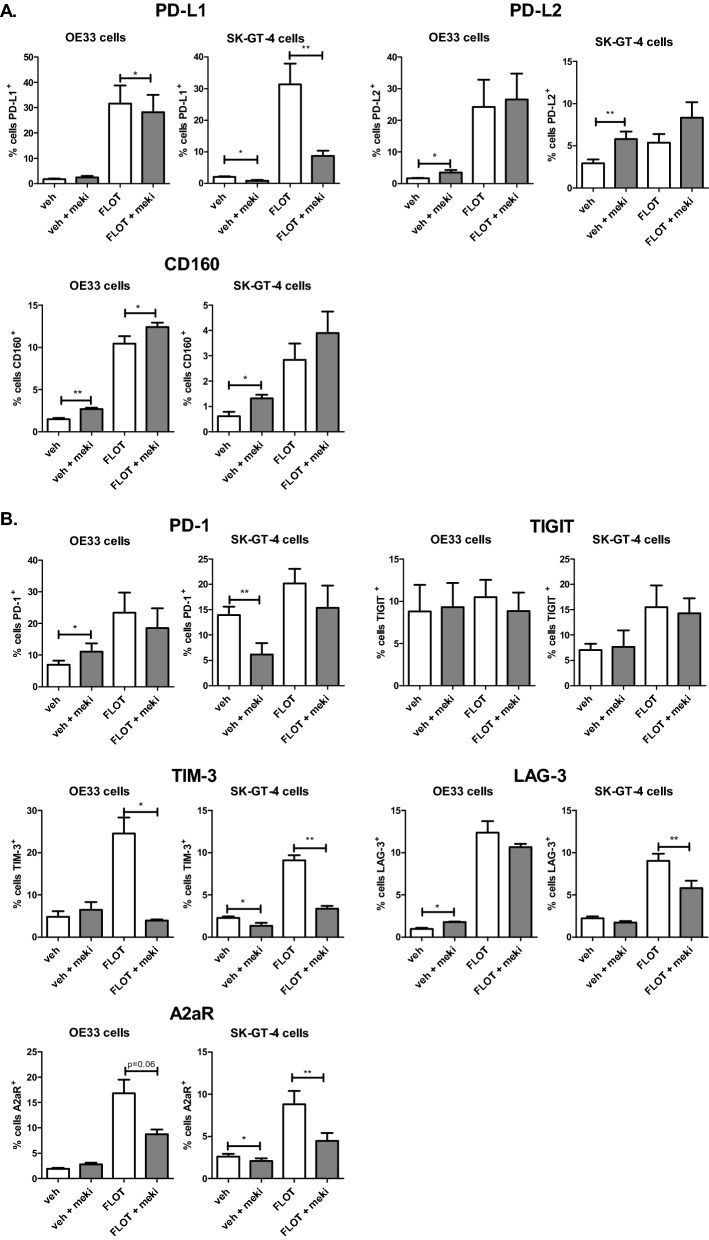
Inhibition of MEK signalling attenuates chemotherapy-induced upregulation of PD-L1, TIM-3, LAG-3 and A2aR on the surface of OAC cells in vitro. OE33 cells and SK-GT-4 cells were treated with and without FLOT chemotherapy regimen in the absence and presence of a MEK inhibitor (meki) for 48 h and the expression of IC ligands (PD-L1, PD-L2 and CD160) and IC receptors (PD-1, TIGIT, TIM-3, LAG-3 and A2aR) on the surface of OAC cells was determined by flow cytometry. Experiments repeated for an n = 5 independent experimental repeats using single technical replicates, Kruskal–Wallis *p < 0.05 and **p < 0.01.

Inhibition of MEK signalling significantly reduced the basal expression of PD-L1 on the surface of SK-GT-4 cells compared with the vehicle control (0.82 ± 0.3 vs. 2.13 ± 0.1%, *p* = 0.02) (Fig. 2A). Moreover, inhibition of MEK signalling significantly decreased FLOT-induced PD-L1 upregulation on the surface of OE33 cells (28.24 ± 6.8 vs. 31.63 ± 7.1%, *p* = 0.03) and SKGT-4 cells (8.75 ± 1.6 vs. 31.38 ± 6.5%, *p* = 0.008) compared with FLOT treatment alone (Fig. 2A).

Inhibition of MEK signalling significantly increased the expression of PD-L2 on the surface of OE33 cells (3.51 ± 0.7 vs. 1.72 ± 0.1%, *p* = 0.02) and SK-GT-4 cells (5.79 ± 0.9 vs. 2.93 ± 0.4%, *p* = 0.001) compared with the vehicle control (Fig. 2A). However, inhibition of MEK signalling did not alter the expression of FLOT-induced PD-L2 expression compared with FLOT treatment alone in either cell line.

In parallel, inhibition of MEK signalling significantly increased the expression of CD160 on the surface of OE33 cells (2.69 ± 0.1 vs. 1.4 ± 0.1%, *p* = 0.002) and SK-GT-4 cells (1.32 ± 0.1 vs. 0.60 ± 0.1%, *p* = 0.04) compared with the vehicle control (Fig. 2A). Moreover, inhibition of MEK signalling in combination with FLOT treatment significantly increased the expression of CD160 on the surface of OE33 cells compared with FLOT treatment alone (3.90 ± 0.8 vs. 2.84 ± 0.6%, *p* = 0.03) compared with the vehicle control (Fig. 2A).

Inhibition of MEK signalling significantly increased the expression of PD-1 on the surface of OE33 cells (11.15 ± 2.5 vs. 6.97 ± 1.3%, *p* = 0.01) compared with the vehicle control (Fig. 2B). In contrast, inhibition of MEK signalling significantly decreased the expression of PD-1 on the surface of SK-GT-4 cells (15.39 ± 4.3 vs. 20.20 ± 2.8%, *p* = 0.001) compared with the vehicle control (Fig. 2B). Inhibition of MEK signalling did not significantly alter the expression of TIGIT on the surface of OE33 or SK-GT-4 cells basally or in combination with FLOT treatment (Fig. 2B).

Inhibition of MEK signalling significantly reduced the expression of basal levels of TIM-3 on the surface of SK-GT-4 cells compared with the vehicle control (1.36 ± 0.3 vs. 2.26 ± 0.2%, *p* = 0.05) (Fig. 2B). Inhibition of MEK signalling significantly decreased FLOT-induced TIM-3 upregulation on the surface of OE33 cells (3.92 ± 0.2 vs. 24.53 ± 3.8%, *p* = 0.03) and SKGT-4 cells (3.35 ± 0.3 vs. 9.09 ± 0.5%, *p* = 0.001) compared with FLOT treatment alone (Fig. 2B).

MEK inhibition significantly increased the expression of basal levels of LAG-3 on the surface of OE33 cells compared with the vehicle control (1.77 ± 0.1 vs. 0.96 ± 0.1%, *p* = 0.02) (Fig. 2B). In contrast, inhibition of MEK signalling significantly decreased FLOT-induced LAG-3 upregulation on the surface of SKGT-4 cells (5.80 ± 0.8 vs. 9.03 ± 0.8%, *p* = 0.002) compared with FLOT treatment alone (Fig. 2B).

Additionally, inhibition of MEK signalling significantly decreased the expression of basal levels of A2aR on the surface of SK-GT-4 cells compared with the vehicle control (2.08 ± 0.3 vs. 2.60 ± 0.3, *p* = 0.04) (Fig. 2B). Also, inhibition of MEK signalling significantly decreased FLOT-induced A2aR upregulation on the surface of SKGT-4 cells (4.47 ± 0.9 vs. 8.80 ± 1.6, *p* = 0.002) compared with FLOT treatment alone (Fig. 2B). In addition, there were a reduction in FLOT-induced A2aR upregulation on the surface of OE33 cells compared with FLOT treatment alone (8.74 ± 0.9 vs. 16.83 ± 2.7, *p* = 0.06, Fig. 2B). To conclude, MEK signalling regulated FLOT-induced upregulation of PD-L1, TIM-3, LAG-3 and A2aR on the surface of OAC cells.

Evidence in the literature demonstrated that STAT3 signalling regulates PD-1 expression on the surface of T cells^38^ and PD-L1 expression on the surface of colon cancer cells^39^, therefore, we hypothesised that STAT3 signalling may regulate PD-1 expression and perhaps other ICs on the surface of OAC cells and assessed if STAT3 inhibition might affect basal expression of tumour-expressed ICs or FLOT-induced upregulation of ICs (Figure S2). Inhibition of the STAT3 signalling pathway had a minimal effect on IC expression in OE33 cells compared with inhibition of the MEK signalling pathway. STAT3 inhibition significantly increased the expression of PD-1 and LAG-3 ICs on OE33 cells basally compared with untreated cells and significantly attenuated FLOT-induced upregulation of A2aR on the surface of OE33 cells compared with FLOT treated OE33 cells (Figure S2). Of note, inhibition of STAT3 signalling had no effect on IC expression in the SK-GT-4 cell line (Figure S2).

### Blockade of PD-L1, PD-1 and A2aR intrinsic signalling in OAC cells enhances the toxicity of the FLOT regimen

**Figure 3:**
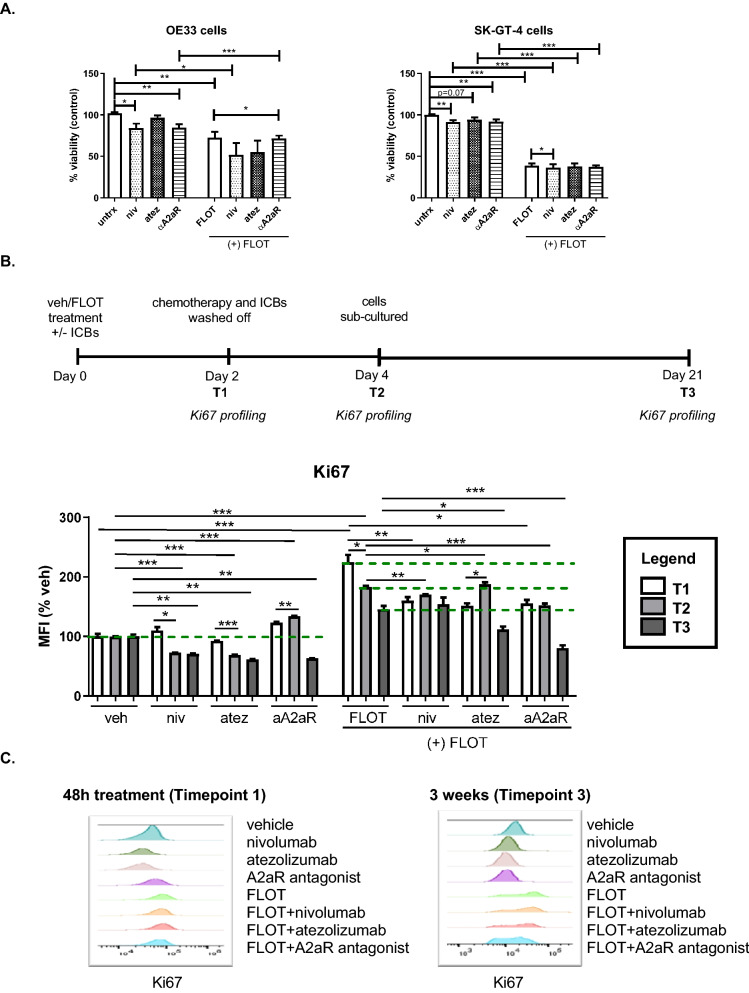
Atezolizumab enhances the toxicity of FLOT chemotherapy regimen in OAC cells demonstrated by a significant decrease in viability and proliferation. (**A**) OE33 cells and SK-GT-4 cells were treated with nivolumab (10 μg/ml), atezolizumab (10 μg/ml) or A2aR antagonist (3 μM) in the absence or presence of FLOT regimen for 48 h and a CCK-8 assay was performed. Experiments were carried out for an n = 4 independent experimental repeats using duplicate technical replicates. (**B**,**C**) OE33 cells were treated with nivolumab (10 μg/ml), atezolizumab (10 μg/ml) or A2aR antagonist (3 μM) in the absence or presence of FLOT regimen for 48 h (timepoint 1: T1), washed twice and allowed to grow for an additional 48 h (T2) after which the cells were sub-cultured in new flasks and left to recover for 3 weeks (T3). Schematic representation of experimental setup depicted in (**B**). (**C**) Ki67 mean fluorescence intensity (MFI) was assessed by intracellular flow cytometry in viable OE33 cells and these experiments were carried out for an n = 4 independent experimental replicates using singlet technical replicates. MFI is expressed as a percentage of vehicle control ± SEM. (**C**) Representative histograms displayed for each treatment at T1 and T3 showing effect of each treatment on Ki67 expression in OE33 cell line. Kruskal–Wallis was used for part (**A**) and a Two-way Anova using Benjamini and Hochberg to correct for false discovery rate was used for part (**C**), *p < 0.05, **p < 0.01 and ***p < 0.001.

Given the observation that the pro-survival MEK signalling pathway regulated FLOT-induced PD-L1 and A2aR upregulation on the surface of OAC cells, we investigated if blockade of PD-1 (nivolumab), PD-L1 (atezolizumab) or A2aR signalling axes in OAC cells might enhance the toxicity of FLOT chemotherapy regimen (Fig. 3).

Single agent nivolumab or A2aR antagonist significantly decreased the viability of OE33 cells compared with untreated cells (84.12 ± 5.2 vs. 102.1 ± 1.4%, *p* = 0.02 and 84.56 ± 4.1 vs. 102.4 ± 1.8%, *p* = 0.003, respectively Fig. 3A). Complementary results were observed in the SK-GT-4 cell line; whereby single agent nivolumab, atezolizumab or A2aR antagonist all decreased the viability of SK-GT-4 cells compared with untreated cells (91.13 ± 2.3 vs. 100.0 ± 0.9%, *p* = 0.004, 94.15 ± 2.8 vs. 100.0 ± 0.9%, *p* = 0.07 and 91.70 ± 2.7 vs. 100.0 ± 0.9%, *p* = 0.008, respectively Fig. 3A).

Combining A2aR antagonist with FLOT significantly decreased the viability of OE33 cells compared with FLOT alone (71.69 ± 3.1 vs. 72.60 ± 7.0%, *p* = 0.03, Fig. 3A). Dual nivolumab and FLOT treatment significantly decreased the viability of SK-GT-4 cells compared with FLOT treatment alone (36.01 ± 4.4 vs. 38.72 ± 2.7%, *p* = 0.01, Fig. 3A).

In summation, single agent nivolumab and A2aR antagonism significantly decreased the viability of OAC cells. Of note, combining nivolumab or A2aR antagonist with the FLOT regimen significantly enhanced the reduction in viability of OAC cells compared with FLOT alone.

Given these findings we sought to investigate how blockade of the PD-1, PD-L1 or A2aR signalling axes alone and in combination with FLOT might affect the proliferation of OE33 cells longitudinally (Fig. 3B). Single agent nivolumab significantly decreased Ki67 expression in OE33 cells at 4 days (72.68 ± 0.2 vs. 100.0 ± 0.4%, *p* < 0.0001) and 21 days (71.07 ± 0.2 vs. 100.0 ± 3.0%, *p* = 0.01) compared with vehicle treated cells (Fig. 3C). Likewise, single agent atezolizumab significantly decreased Ki67 expression in OE33 cells 4 days (61.34 ± 0.4 vs. 100.0 ± 3.0%, *p* < 0.0001) and 21 days (61.34 ± 0.4 vs. 100.0 ± 3.0%, *p* = 0.005) compared with the vehicle (Fig. 3C). Single agent A2aR antagonist significantly increased Ki67 expression in OE33 cells at 4 days (139.9 ± 0.3 vs. 100.0 ± 3.0%, *p* < 0.0001) and decreased Ki67 expression at 21 days (63.68 ± 0.2 vs. 100.0 ± 3.0%, *p* = 0.007) compared with vehicle treated cells (Fig. 3C).

Furthermore, 48 h FLOT treatment significantly increased Ki67 in OE33 cells compared with the vehicle at 2 days (224.4 ± 12.4 vs. 100.0 ± 4.0%, *p* = 0.0008), 4 days (183.3 ± 1.8 vs. 100.0 ± 0.4%, *p* < 0.001) and 21 days (145.95 ± 5.2 vs. 100.0 ± 3.0%, *p* = 0.002) (Fig. 3C). However, Ki67 expression was significantly decreased in the FLOT-treated cells 21 days post-treatment compared with FLOT treated cells 4 days post-treatment (145.95 ± 5.2 vs. 183.3 ± 1.8 vs. 100.0 ± 0.4%, *p* = 0.01) and compared with FLOT-treated cells 2 days post-treatment (145.95 ± 5.2 vs. 224.4 ± 12.4%, *p* = 0.03) (Fig. 3C).

Single agent nivolumab in combination with FLOT treatment significantly decreased Ki67 expression in OE33 cells compared with FLOT treatment alone 2 days post-treatment (159.8 ± 6.1 vs. 224.4 ± 12.36%, *p* = 0.005) and 4 days post-treatment (170.3 ± 0.4 vs. 183.3 ± 1.8%, *p* = 0.003) (Fig. 3C). Equally, single agent atezolizumab in combination with FLOT treatment significantly decreased Ki67 expression in OE33 cells compared with FLOT treatment alone 2 days post-treatment (151.9 ± 3.7 vs. 224.4 ± 12.36%, *p* = 0.02) and 21 days post-treatment (112.2 ± 4.6 vs. 145.9 ± 5.2%, *p* = 0.02) (Fig. 3C).

Single agent A2aR antagonist in combination with FLOT treatment significantly decreased Ki67 expression in OE33 cells compared with FLOT treatment alone 2 days post-treatment (155.8 ± 5.8 vs. 224.4 ± 12.36%, *p* = 0.02), 4 days post-treatment (152.7 ± 2.5 vs. 183.3 ± 1.8%, *p* = 0.0001) and 21 days post-treatment (80.49 ± 4.6 vs. 145.9 ± 5.2%, *p* = 0.0007) (Fig. 3C).

In conclusion, single agent nivolumab, atezolizumab and A2aR antagonism significantly decreased the proliferation of OAC cells alone. Strikingly, combining single agent nivolumab, atezolizumab and A2aR antagonism with the FLOT regimen significantly decreased the proliferation of OAC cells compared with FLOT treatment. Taken together these findings suggest that inhibition of the PD-1 axis or A2aR axis decreases the proliferation of OAC cells and when combined with the FLOT regimen synergistically enhance the toxicity of FLOT against OAC cells in vitro.

**Figure 4:**
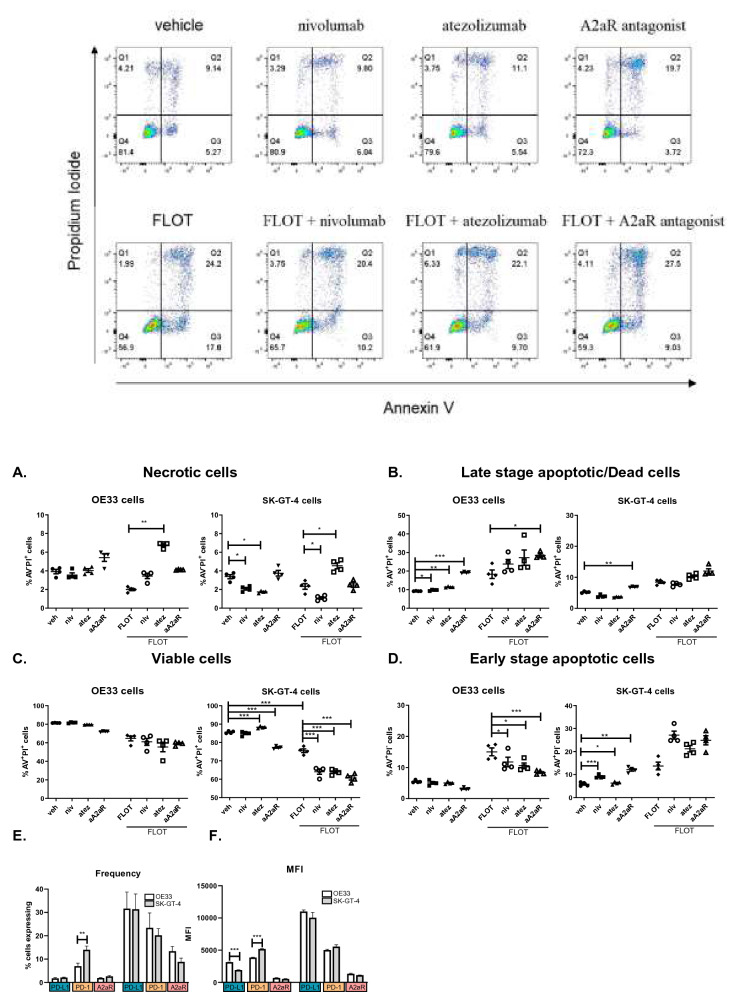
PD-1, PD-L1 and A2aR blockade enhances the toxicity of FLOT chemotherapy regimen demonstrated by a significant reduction in the percentage of viable cells and increase in late stage apoptotic/dead cells. OE33 cells and SK-GT-4 cells were treated with nivolumab (10 μg/ml), atezolizumab (10 μg/ml) or A2aR antagonist (3 μM) in the absence or presence of FLOT regimen for 48 h. Viability was determined by flow cytometry using annexin V propidium iodide assay. Necrotic cells ((**A**) AV^−^PI^+^), late stage apoptotic/dead cells ((**B**) AV^+^PI^+^), viable cells ((**C**) AV^−^PI^−^) and early stage apoptotic cells ((**D**) AV^+^PI^−^), dead were characterised. Representative dot plots shown for each treatment in OE33 cell line. Experiments repeated for n = 4 independent experimental repeats using singlet technical replicates. Kruskal–Wallis used for part (**A**–**D**) and Mann–Whitney test used for part (**E**,**F**), *p < 0.05, **p < 0.01 and ***p < 0.001.

In light of these findings, we next investigated how blockade of PD-1, PD-L1 or A2aR signalling axes alone and in combination with FLOT might affect OAC cell apoptosis and cell death (Fig. 4). Dual atezolizumab and FLOT treatment significantly increased the percentage of necrotic OE33 cells compared with FLOT treatment alone (Fig. 4A). Single agent nivolumab or atezolizumab treatment significantly reduced the percentage of necrotic SK-GT-4 cells compared with the vehicle (Fig. 4A). Combining nivolumab with FLOT significantly decreased the percentage of necrotic SKG-GT-4 cells compared with FLOT treatment alone and combining atezolizumab with FLOT treatment significantly increased the percentage of necrotic SK-GT-4 cells compared with FLOT treatment alone (Fig. 4A).

Single agent nivolumab, atezolizumab or A2aR antagonist significantly induced OE33 cell death/late stage apoptosis compared with the vehicle control (Fig. 4B). Comparably, single agent A2aR antagonist significantly induced cell death/late stage apoptosis in SK-GT-4 cells (6.88 ± 0.1 vs. 5.21 ± 0.1%, *p* = 0.001) compared with the vehicle control (Fig. 4B). In addition, single agent nivolumab (9.13 ± 0.3 vs. 5.8 ± 0.4%, *p* = 0.005) and A2aR antagonist (12.10 ± 0.6 vs. 5.8 ± 0.4%, *p* = 0.0004) significantly increased the percentage of early stage apoptotic SK-GT-4 cells compared with untreated cells (Fig. 4D).

Combining single agent nivolumab, atezolizumab or A2aR antagonist with FLOT significantly increased the percentage of early stage apoptotic OE33 cells compared with FLOT treatment (Fig. 4D). However, in parallel there were trends toward an increase in the percentage of late stage apoptotic/dead OE33 cells following the dual combination of nivolumab or atezolizumab with FLOT treatment compared with FLOT alone (Fig. 4B). And a significant increase in late stage apoptotic/dead OE33 cells was observed following dual A2aR antagonist and FLOT compared with FLOT treatment (Fig. 4B). Collectively, the reduction in the frequency of early stage apoptotic OE33 cells and concomitant induction of late stage apoptotic/dead OE33 cells following dual ICB-FLOT treatment might reflect an increase in the induction of apoptotic-induced cell death at this timepoint.

Analogously, combining single agent atezolizumab (10.28 ± 0.4 vs. 8.44 ± 0.3%, *p* = 0.04) or A2aR antagonist (12.00 ± 0.7 vs. 8.44 ± 0.3%, *p* = 0.04) with FLOT significantly induced late stage apoptotic/cell death in SK-GT-4 cells compared with FLOT (Fig. 4B). Moreover, single agent atezolizumab and A2aR antagonist significantly decreased the percentage of viable SK-GT-4 cells compared with the vehicle (Fig. 4C). In conjunction, dual nivolumab, atezolizumab or a2aR antagonist with FLOT treatment significantly decreased the percentage of viable SK-GT-4 cells compared with FLOT (Fig. 4C).

Although, combining single agent nivolumab with FLOT did not significantly enhance SK-GT-4 cell death, a significant increase in early stage apoptotic SK-GT-4 cells was observed using combination nivolumab with FLOT compared with FLOT alone (27.15 ± 1.7 vs. 13.73 ± 1.6%, *p* = 0.0002) (Fig. 4D). Furthermore, combining single agent atezolizumab (27.15 ± 1.7 vs. 13.73 ± 1.6%, *p* = 0.001) or A2aR antagonist (24.90 ± 1.9 vs. 13.73 ± 1.6%, *p* = 0.0003) with FLOT significantly induced increased the percentage of early stage apoptotic SK-GT-4 cells compared with FLOT treated cells (Fig. 4D). Total cell counts of viable, necrotic, early stage apoptotic and late stage apoptotic/dead cells were also assessed and shown in Figure S3 which reflected similar results to Fig. 4 which depicted the frequency of those cell types.

These findings highlight that single agent PD-1, PD-L1 and A2aR IC blockade induced apoptosis and OAC cell death. Furthermore, combining ICB with the FLOT chemotherapy regimen synergistically enhanced induction of apoptosis in OAC cells and OAC cell death.

Given the stark differences in ICB toxicity between both cell lines we also compared the frequency and MFI of IC expression between the two cell lines (Fig. 4E-F). Of note the SK-GT-4 cell line expressed significantly higher frequencies (13.97 ± 1.6 vs. 6.97 ± 1.3%, *p* = 0.0001) and MFI levels (5184 ± 61.4 vs. 3867 ± 50.0%, *p* = 0.0001) for PD-1 compared with the OE33 cell line, which may explain the enhanced toxicity of nivolumab treatment against the SK-GT-4 cell line (Fig. 4E-F). Although the frequency of PD-L1 expression was comparable between the OE33 cell line and the SK-GT-4 cell line, the OE33 cells did have significantly higher MFI levels for PD-L1 compared with the SK-GT-4 cell line basally (3142 ± 8.9 vs. 1924 ± 27.6%, *p* = 0.005) (Fig. 4E-F). In this context, single agent atezolizumab significantly induced cell death/late stage apoptosis in OE33 cells only and not in the SK-GT-4 cell line. Therefore, differences in the basal expression MFI levels of PD-1 and PD-L1 between the OE33 and SK-GT-4 cell lines may explain the observed differences in toxicity for single agent nivolumab and atezolizumab. A2aR frequency and MFI levels were comparable between both cell lines (Fig. 4E-F), and as such the expression levels of A2aR does not offer a potential explanation in the observed differences in sensitivity to A2aR antagonism between the two cell lines.

### Blockade of IC signalling in OAC cells decreases the formation of γH2AX and expression of DNA repair genes

**Figure 5:**
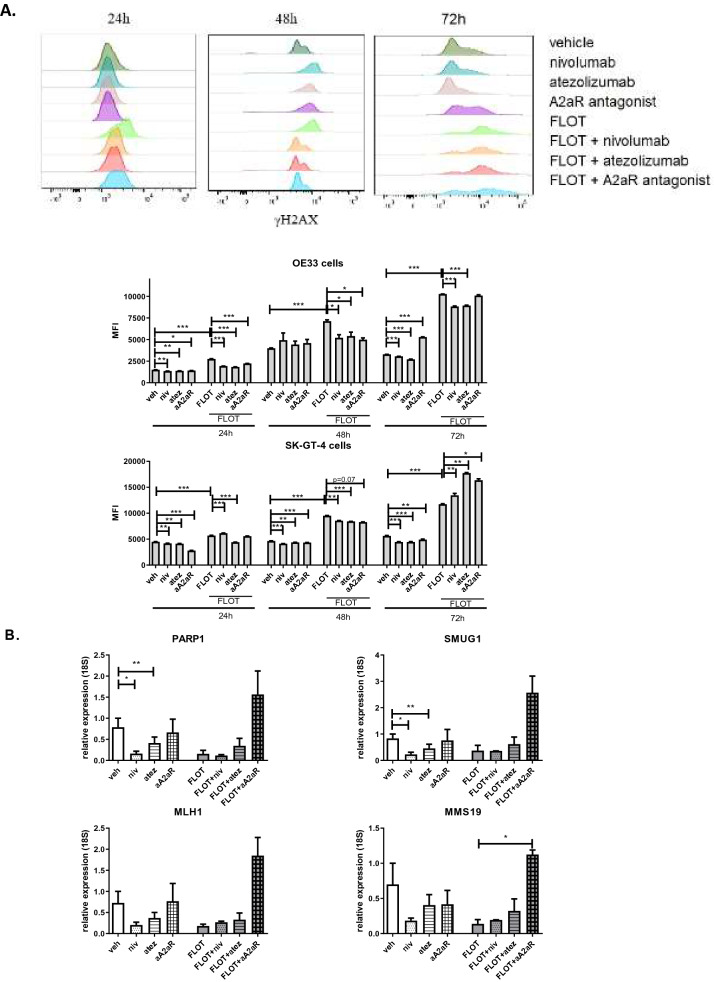
Single agent nivolumab and atezolizumab decreased the levels of γH2AX and the levels of DNA repair genes in vitro*.* (**A**) OE33 cells and SK-GT-4 cells were treated with nivolumab (10 μg/ml), atezolizumab (10 μg/ml) or A2aR antagonist (3 μM) in the absence or presence of FLOT regimen for 24 h, 48 h and 72 h. Expression of γH2aX was determined by intracellular flow cytometry. γH2ax expression is presented as MFI. Representative histograms showing the levels of γH2AX in OE33 cells for each treatment at 24 h, 48 h and 72 h. Experiments repeated for an n = 4 independent experimental repeats using singlet technical replicates. (**B**) SK-GT-4 cells were treated with nivolumab (10 μg/ml), atezolizumab (10 μg/ml) or A2aR antagonist (3 μM) in the absence or presence of FLOT regimen for 48 h. mRNA expression levels of *PARP1, SMUG1, MMS19* and *MLH1* were determined by qPCR. qPCR experiments were conducted for an n = 3 independent experimental replicates in triplicate technical replicates. Expression presented as relative quantity of *18S* housekeeping gene. Two-way Anova using Benjamini and Hochberg to correct for false discovery rate in part (**A**) and Kruskal–Wallis used in part (**B**) *p < 0.05 and **p < 0.01, ***p < 0.001.

We have shown that PD-1, PD-L1 and A2aR signalling confers OAC cells with a survival advantage as their blockade alone reduces OAC cell viability and can enhance FLOT chemotherapy toxicity. Of relevance, studies have implicated a role for PD-L1 intrinsic signalling in mediating DNA repair in colon cancer^40^. Therefore, to achieve a greater understanding of the mechanisms of action behind enhanced FLOT cytotoxicity in combination with ICB we assessed if blockade of these IC pathways might alter the formation of γH2AX alone and in combination with FLOT chemotherapy (Fig. 5). Tumour cells rapidly proliferate and typically acquire DNA damage during replication generating genotoxic stress in the cells, which ultimately leads to tumour cell death if left unrepaired. Formation of γH2AX is an important step in the initiation of DNA repair.

Single agent nivolumab, atezolizumab and A2aR antagonist significantly decreased the levels of γH2AX expression in OE33 cells following 24 h treatment compared with the vehicle control (nivolumab: 1352 ± 15.09 vs. 1507 ± 8.51%, *p* = 0.005, atezolizumab: 1383 ± 6.8 vs. 1507 ± 8.51%, *p* = 0.002 and A2aR antagonist: 1416 ± 13.6 vs. 1507 ± 8.51%, *p* = 0.02) (Fig. 5A). Analogous findings were observed in the SK-GT-4 cell line where single agent nivolumab, atezolizumab and A2aR antagonist significantly decreased the levels of γH2AX expression following 24 h compared with the vehicle control (nivolumab: 4167 ± 50.85 vs. 4491 ± 32.9%, *p* = 0.001, atezolizumab: 4129 ± 33.1 vs. 4491 ± 32.9%, *p* = 0.002 and A2aR antagonist: 2791 ± 38.6 vs. 4491 ± 32.9%, *p* < 0.0001) (Fig. 5A).

Following 24 h treatment with FLOT the levels of γH2AX expression in OE33 cells was significantly increased compared with the vehicle control (2756 ± 29.05 vs. 1507 ± 8.5%, *p* < 0.0001). Additionally, in the SK-GT-4 cell line following 24 h treatment with FLOT the levels of γH2AX expression were significantly increased compared with the vehicle control (5694 ± 49.6 vs. 4491 ± 32.9%, *p* = 0.0001) (Fig. 5A).

Combining single agent nivolumab, atezolizumab and A2aR antagonist with the FLOT regimen significantly decreased the levels of γH2AX expression in OE33 cells following 24 h compared with FLOT treated cells (nivolumab: 1937 ± 9.9 vs. 2756 ± 29.0%, *p* = 0.0001, atezolizumab: 1836 ± 12.1 vs. 2756 ± 29.0%, *p* = 0.0001 and A2aR antagonist: 2232 ± 11.8 vs. 2756 ± 29.0%, *p* = 0.0002) (Fig. 5A). In the same way, combining single agent atezolizumab with the FLOT regimen significantly decreased the levels of γH2AX expression in SK-GT-4 cells following 24 h compared with FLOT treated cells (4414 ± 13.1 vs. 5694 ± 49.6%, *p* < 0.0001) (Fig. 5A). On the contrary, combining single agent nivolumab with the FLOT regimen significantly increased the levels of γH2AX expression in SK-GT-4 cells following 24 h compared with FLOT alone (nivolumab: 6138 ± 55.2 vs. 5694 ± 49.6%, *p* < 0.0001) (Fig. 5A).

Likewise, these trends were similarly observed at 48 h and 72 h whereby single agent nivolumab, atezolizumab and A2aR antagonism decreased γH2AX expression in SK-GT-4 cells compared with the vehicle control (Fig. 5A). In OE33 cells at a 72 h timepoint, although single agent nivolumab and atezolizumab significantly decreased γH2AX expression in OE33 cells compared with the vehicle control A2aR antagonism significantly increased γH2AX expression in OE33 cells compared with the vehicle control (Fig. 5A).

Furthermore, at 48 h combining single agent nivolumab, atezolizumab and A2aR antagonist with the FLOT regimen significantly decreased the levels of γH2AX expression in OE33 cells compared with FLOT treated cells. At 72 h, the dual combination of nivolumab or atezolizumab with FLOT significantly decreased γH2AX expression in OE33 cells compared with FLOT treatment alone (Fig. 5A). At a 48 h timepoint in SK-GT-4 cells, combining single agent nivolumab or atezolizumab with FLOT significantly decreased γH2AX expression compared with FLOT only. Moreover, the combination of A2aR antagonist with FLOT decreased γH2AX expression compared with FLOT treatment alone at the 48 h timepoint (Fig. 5A). However, at 72 h combining ICB with FLOT treatment increased γH2AX expression in SK-GT-4 cells compared with FLOT treated cells (Fig. 5A).

Furthermore, Tu et *al*.^41^, demonstrated that intracellular PD-L1 acts as an RNA binding protein enhancing the mRNA stability of NBS1 and BRCA1, thus upregulating the expression of DNA repair proteins NBS1 and BRCA1. Therefore, we assessed if ICB might alter the expression of well described DNA repair genes. We selected the DNA repair genes *PARP1, SMUG1, MLH1* and *MMS19* to include in our analyses as these genes were previously found to be upregulated in OAC patients who had a poor subsequent response to first-line chemoradiotherapy treatment^42–44^. Therefore, this study assessed the effect of ICB alone and in combination with FLOT chemotherapy on the expression of these 4 DNA repair genes using the SK-GT-4 cells as a model (Fig. 5). Single agent atezolizumab and nivolumab significantly reduced the mRNA expression levels of *PARP1* and *SMUG1* compared with the vehicle control (*PARP1*: 0.41 ± 0.1 and 0.22 ± 0.1 vs. 0.78 ± 0.2%, *p* = 0.005 and *p* = 0.04 and *SMUG1*: 0.45 ± 0.1 and 0.45 ± 0.1 vs. 0.83 ± 0.1%, *p* = 0.008 and *p* = 0.04) (Fig. 5B). Combining A2aR antagonist with the FLOT regimen significantly increased the mRNA expression levels of *MMS19* compared with FLOT treated cells (1.12 ± 0.1 vs. 0.13 ± 0.06%, *p* = 0.03) (Fig. 5B).

Taken as a whole, single agent nivolumab, atezolizumab and A2aR antagonist decreased γH2AX expression in both OE33 and SK-GT-4 cell lines at 24 h and 48 h. However, at a 72 h timepoint A2aR antagonist differentially increased and decreased γH2AX expression in OE33 cells and SK-GT-4 cells, respectively. Combining ICB with FLOT significantly decreased γH2AX expression in OE33 cells at each timepoint. However, in SK-GT-4 cells differential effects were observed at the different timepoints in which the dual ICB-FLOT combination decreased γH2AX expression at 48 h but then increased its expression at 72 h. Therefore, we subsequently assessed what effect these drugs had on expression of DNA repair genes at 48 h in the SK-GT-4 cell line. We observed that single agent atezolizumab decreased the expression of *PARP1* and *SMUG1* DNA repair genes, in parallel with a concomitant decrease in γH2AX expression levels at the 48 h timepoint. Furthermore, combination FLOT and PD-1/PD-L1 ICB did not significantly alter the expression of DNA repair genes in SK-GT-4 cells. However, although dual A2aR antagonist in combination with FLOT decreased γH2AX expression in SK-GT-4 cells at 48 h, an increase in expression of DNA repair genes was observed at 48 h in the SK-GT-4 cell line.

### Blockade of PD-1 axis signalling in OAC cells decreases aldehyde dehydrogenase (ALDH) stem-like marker

**Figure 6:**
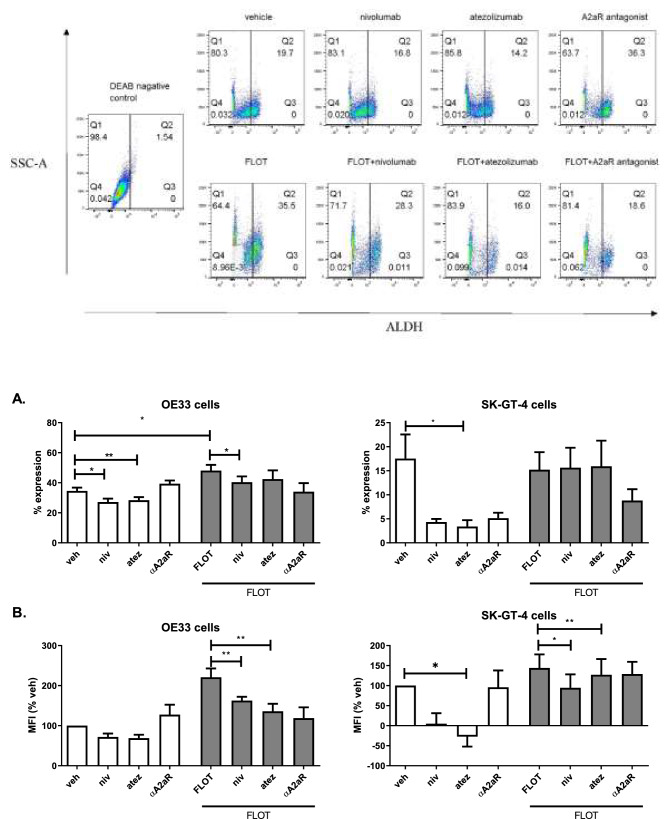
Nivolumab and atezolizumab treatment decrease the percentage of ALDH^+^ stem-like OAC cells in vitro. OE33 cells and SK-GT-4 cells were treated with nivolumab (10 μg/ml), atezolizumab (10 μg/ml) or A2aR antagonist (3 μM) in the absence or presence of the FLOT regimen for 48 h. ALDH activity was determined using an aldefluor assay by flow cytometry. Representative dot plots shown for each treatment and for the DEAB negative control which was used to assess baseline fluorescence to allow accurate gating for ALDH activity. (**A**) depicts the frequency of ALDH + OAC cells and (**B**) depicts the median fluorescence intensity of ALDH as a percentage of the vehicle control. Experiments repeated for an n = 4 independent experimental repeats using singlet technical replicates. *p < 0.05 and **p < 0.01, Kruskal–Wallis statistical test.

Cancer stem-like cells exist as part of a subpopulation within tumours and are thought to be a major contributor to tumour recurrence. Moreover, our findings demonstrate that ICB decreases OAC cell proliferation and viability and induced OAC cell apoptosis and cell death in a subpopulation of OAC cells. Therefore, we aimed to investigate if ICB might be targeting the stem-like compartment within a population of OAC cells so we assessed the effect of ICB alone and in combination with FLOT on the levels of aldehyde dehydrogenase (ALDH) activity, which is a recognised marker of stem-like OAC cells^45^ (Fig. 6). A study previously published by our group demonstrated that the FLOT regimen preferentially upregulated PD-L1 on the surface of ALDH^+^ stem-like OAC cells and PD-1 on the surface of ALDH^−^ non-stem-like associated OAC cells^35^.

We found that single agent nivolumab and single agent atezolizumab significantly decreased the frequency of ALDH^+^ OE33 cells compared with the vehicle control (27.17 ± 2.2 (*p* = 0.02) vs. 28.37 ± 2.1 (*p* = 0.001) and 34.57 ± 2.1%, respectively) (Fig. 6A). However, FLOT chemotherapy treatment significantly increased the frequency of ALDH^+^ OE33 cells compared with the vehicle control (48.12 ± 3.7 vs. 34.57 ± 2.1%, *p* = 0.01) (Fig. 6A). Interestingly, nivolumab significantly attenuated the FLOT-induced increase in the frequency of ALDH^+^ OE33 cells compared with FLOT treated cells (38.13 ± 4.0 vs. 48.12 ± 3.7%, *p* = 0.02) (Fig. 6A). In addition, single agent nivolumab and atezolizumab significantly decreased ALDH MFI compared with the vehicle control (Fig. 6B).

In the SK-GT-4 cell line, we observed a significant decrease in the frequency of ALDH^+^ cells following single agent atezolizumab treatment compared with the vehicle control in SK-GT-4 cells (3.39 ± 1.3 vs. 12.11 ± 4.8%, *p* = 0.08) (Fig. 6A). Similarly, single agent atezolizumab significantly decreased ALDH MFI in SK-GT-4 cells compared with untreated cells (Fig. 6B). Although ICB did not significantly decrease the frequency of ALDH^+^ SK-GT-4 cells, we did observed that single agent nivolumab and atezolizumab in combination with FLOT treatment significantly decreased ALDH MFI compared with FLOT alone (Fig. 6B).

To summarise, PD-L1 blockade decreased the expression of ALDH stem-like marker in OAC cells and PD-1 blockade attenuated the FLOT-induced increase in ALDH activity in OE33 cells but not SK-GT-4 cells.

In conclusion, PD-L1 and PD-1 blockade decreased the expression of ALDH stem-like marker in OE33 cells and only PD-L1 blockade decreased ALDH stem-like marker in SK-GT-4 cells. Both PD-1 blockade and PD-L1 blockade attenuated the FLOT-induced increase in ALDH activity in OE33 cells and SK-GT-4 cells.

## Discussion

We and others have previously shown that tumour cells express an array of both IC ligands and IC receptors^35^. Other studies have also demonstrated that tumour cells express an array of IC receptors such as TIM-3, A2aR and PD-1 on their cell surfaces as well as ligands such as PD-L1 and PD-L2^15,17,18,20,46–50^. Of particular clinical relevance we have previously shown that the FLOT chemotherapy regimen increases the expression of an array of ICs on the surface of OAC cells driving an immune-resistant phenotype within the tumour^35^. Furthermore, tumour cell-expressed ICs have also been implicated as a potential mechanism of chemo(radio)-resistance in a range of cancer types^15,17,18,20,46–50^. Firstly, by immune-dependent pathways via binding of ICs expressed on tumour cells with their cognate receptors or ligands on tumour-infiltrating immune cells negatively regulating anti-tumour function of these infiltrating immune cells^51^. Chemotherapy-induced PD-L1 upregulation on the surface of breast cancer cells results in immune evasion via ligation of PD-L1 to PD-1 on T cells, thereby inducing T cell apoptosis^51^. Alternatively, immune-independent mechanisms mediated by ICs have recently been recognised as potential mechanisms of resistance employed by tumour cells to attenuate the efficacy of chemo(radio)therapy^15,17,18,20,46–50^. Such mechanisms include promotion of key hallmarks of cancer via IC-intrinsic signalling in tumour cells including tumour cell metastasis^15^, enhanced DNA repair^18^, tumour cell proliferation^20^, cancer stem-like activity^17^ and glycolysis^50^ in tumour cells. Intriguingly, in this study following recovery of the tumour cells post-FLOT treatment TIM-3, LAG-3 and A2aR expression on the surface of OE33 cells was significantly decreased compared with the vehicle control. These findings recapitulate observations from a previous study by our group which demonstrated that the expression of TIM-3, LAG-3 and A2aR was significantly decreased on the surface of tumour cells in OAC tumour tissue biopsies post-FLOT treatment^35^.

The findings from this study demonstrated that signalling through RAS/RAF/MEK/ERK (MAPK) signalling cascade upregulated several IC proteins including PD-L1, TIM-3, LAG-3 and A2aR on the surface of OAC cells following FLOT treatment. A complementary study demonstrated that MAPK signalling regulated epidermal growth factor- and interferon-gamma-induced PD-L1 expression in lung adenocarcinoma cells and that inhibition of MEK1/2 attenuated PD-L1 upregulation^29^. MEK signalling has pleiotropic effects in enhancing fundamental pro-tumourigenic processes, including tumour cell growth, survival and differentiation^52^. Collectively, these findings support a rationale for combination MAPK signalling and PD-L1 blockade to boost anti-tumour immunity as MAPK-induced upregulation of novel ICs such as TIM-3, LAG-3 and A2aR might represent mechanisms of immune escape or acquired resistance to PD-L1 ICB. A study in murine models further supports this rationale as dual MAPK inhibition in combination with anti-PD-L1 ICB resulted in synergistic and durable tumour regression even where either agent alone was only modestly effective^53^. The combination promoted T cell anti-tumour activity in combination with PD-L1 ICB^53^. Although MEK inhibition did profoundly block naive CD8^+^ T cell priming, an increased number of tumour-infiltrating, effector-phenotype, antigen-specific CD8^+^ T cells was observed^53^. Furthermore, MEK inhibition protected tumour-infiltrating CD8^+^ T cells from chronic TCR stimulation-induced cell death while sparing cytotoxic activity^53^. MEK inhibition mediated downregulation of ICs on tumour cells may have also contributed to these synergistic effects.

This study also profiled the dynamic alterations in the IC expression profiles of OAC cells post-FLOT treatment until complete recovery of OAC cells. Markedly IC expression was significantly upregulated in the immediate days post-FLOT treatment and was sustained longitudinally for up to 3 weeks on the surface of tumour cells. Upon complete recovery and subculture of tumour cells, we found that although PD-L1 expression had reduced substantially, expression remained significantly higher on the surface of post-FLOT tumour cells compared with untreated cells. Chemotherapy has been shown to select for a more treatment resistant tumour cell phenotype. Of particular clinical relevance, an isogenic model of cisplatin resistant lung cancer cells displayed significantly higher levels of PD-L1 expression compared with matched cisplatin sensitive cells^54^. Therefore, considering the findings of this study in context with the wider literature, the increased expression of PD-L1 on the surface of recovered OAC cells may suggest that PD-L1 expression may identify but also confer a more treatment resistant phenotype.

PD-L1 upregulation is associated with activation of the DNA double-strand break repair pathway in patients with colon cancer^40^. Furthermore, several studies have shown that accumulation of damaged DNA and subsequent DNA damage signalling mediates upregulation of PD-L1 on the surface of tumour cells^18,55,56^. In turn, additional studies have identified a role of PD-L1 tumour cell intrinsic signalling in enhancing the DNA damage response^57^. Tu et *al*.^41^, discovered that intracellular PD-L1 acts as an RNA binding protein that regulates the mRNA stability of NBS1 and BRCA1, thus enhancing repair of damaged DNA. In light of the mounting evidence that PD-L1 has a positive feedback regulatory role in the DNA damage response in cancer, our complementary findings further consolidate this novel immune-independent role in OAC. We demonstrated that inhibition of the PD-1 signalling axis in OAC cells decreased expression of γH2AX, a surrogate marker of DNA damage which plays an important role in the DNA damage signalling response and subsequent repair of damaged DNA^58^, and inhibited expression of DNA repair genes PARP1 and SMUG1 alone and in combination with FLOT.

Following FLOT treatment there was a higher percentage of ALDH^+^ OAC cells, consistent with previously published findings from our group^35^. This increase in ALDH activity may reflect enrichment of FLOT chemoresistant clones that are perhaps positive for stem-like markers, such as ALDH. Another possibility is that tumour cells may have increased their ALDH activity in response to FLOT treatment in an attempt to adapt and survive the adverse conditions of chemotherapy treatment. It is plausible and most likely that both scenarios are occurring in tandem as we observed a significant increase in both the frequency of cells that are positive for ALDH activity and a significant increase in the absolute levels of ALDH activity post-FLOT treatment.

The expression of A2aR on a sub-fraction of OAC cells might suggest that this receptor is preferentially expressed on rare subpopulations of cancer stem-like cells. Previous studies have shown that A2aR is expressed on the surface of a subpopulation of gastric cancer stem-like cells in gastric adenocarcinoma^59^ which has been shown by TCGA studies to closely resemble OAC on the molecular level^60^. Cancer stem-like cells often comprise a very low percentage of the entire cell population however, the low abundance of cancer stem-like cells does not translate to a lack of clinical importance as this small population of cancer stem-like cells often survive conventional chemotherapy regimens giving rise to tumour recurrence and metastasis. The striking effects of A2aR blockade on viability, proliferation and OAC cell survival points toward an important role for this receptor in OAC cell biology which warrants further investigation regarding a potential role in maintenance of cancer stem-like features and survival. Although we did not observe significant effects of A2aR blockade on ALDH activity, there likely exists other cancer stem-like markers that are important in OAC that have yet to be elucidated, therefore, this does not rule out that A2aR signalling might play an important role in maintaining the cancer stem-like compartment.

Furthermore, elements of the PD-1 signalling axis namely the PD-1 receptor and its cognate ligand PD-L1 have been identified on the surface of stem-like tumour cells in melanoma^61^ and lung cancer^17^. We have previously shown that FLOT upregulates PD-L1 preferentially on the surface of ALDH^+^ stem-like OAC cells^35^. In contrast, the findings from that study demonstrated that PD-1 was preferentially expressed on the surface of ALDH^−^ non-stem-like associated OAC cells^35^. Moreover, PD-1 and PD-L1 tumour cell intrinsic signalling has been shown to promote stem-like characteristics in both melanoma and lung cancer cells^17,61^. The findings of this study demonstrated that blockade of the PD-1 signalling axis decreased ALDH stem-like marker in OAC cells. Collectively, these findings highlight an immune-independent role for the PD-1 axis signalling cascade in driving a treatment resistant phenotype, as cancer stem-like cells are thought to play a pivotal role in resistance to first-line chemotherapy regimens and subsequent tumour recurrence^62,63^. Given that our previous study showed that PD-1 was preferentially expressed on ALDH^−^ non-stem-like associated OAC cells and that blockade of PD-1 signalling in OAC cells in this study demonstrated a decrease in ALDH stem-like activity. This may suggest a potential role for PD-1 intrinsic signalling in ALDH^−^ cells in supporting the maintenance or survival of the ALDH^+^ stemlike compartment perhaps through paracrine signalling or simply via offering a ligating activation signal for PD-L1 expressed on neighbouring ALDH^+^ stem-like OAC cells, which then promotes a stem-like phenotype in said cell. To re-iterate, the effects of nivolumab in reducing stem-like marker expression may be ascribed to a reduced activation of PD-L1 intrinsic signalling in ALDH^+^ stem-like OAC cells due to nivolumab-mediated disruption of PD-1 binding to PD-L1 on ALDH^+^ stem-like OAC cells in a paracrine manner.

Although FLOT induced apoptosis within 48 h and decreased cellular viability we also observed that FLOT increased the proliferation of OAC cells. However, the observed FLOT-induced proliferation of OAC cells was diminished longitudinally. Although this initial increase in proliferation was surprising, the FLOT regimen may collectively hinder DNA damage signalling/repair and subsequent cell cycle arrest or may prevent delays through the cell cycle initially. This may possibly cause the cells to undergo cell cycling at a faster rate, increasing their proliferation at first upon treatment initiation, which then deceases longitudinally as the FLOT-treated cells die off in culture, likely owing to the acquisition of increased cellular genotoxic stress and ROS levels induced by this cocktail of chemotherapies.

There are a number of studies in the literature highlighting that PD-1, PD-L1 and A2aR intrinsic signalling in tumour cells also promote tumour cell proliferation in a range of cancer types including hepatocellular carcinoma^19^, lung, melanoma^20^, ovarian^20^, pancreatic^64^, gastric^15,16,65^ and cervical cancer^16^. The findings of this study further substantiate the immune-independent role of PD-1, PD-L1 and A2aR tumour cell-intrinsic signalling in promoting proliferation of tumour cells in the context of OAC.

The differences observed between both the OE33 and SK-GT-4 cell lines may be attributed to their differential doubling times, the OE33 cell line has a 33 h doubling time and the SK-GT-4 cell line has a longer doubling time of 39-41 h. The OE33 cell line demonstrated the most consistent results uncovering the direct cytotoxic effects of ICB on tumour cells and appeared to be more sensitive to the effects of ICB. The SK-GT-4 cell line did not always show the same sensitivity to ICB regimens and this may be a reflection of the longer doubling time of this cell line and the fact that ICBs may require more time to induce profound cytotoxic or molecular effects, which were observed with the OE33 cell line regarding the effects on the expression of stem-like marker ALDH. The longitudinal effects of ICB on the proliferation status and expression of γH2AX in both cell lines was also assessed at longer timepoints of 48 h and 72 h. To conclude, these findings highlighted that at a longer 72 h timepoint ICB in combination with FLOT increased expression of γH2AX in the SK-GT-4 cell line however at 48 h we observed a decrease in expression. Typically, at longer timepoints of 72 h measuring the rate at which γH2AX is resolved can indicate of how quickly the cell can repair its DNA^58^. Therefore, these findings may suggest that combining ICB with the FLOT regimen may be attenuating or slowing down the DNA repair process and provide a rationale for the build-up of γH2AX expression with the dual combination compared with FLOT alone at 72 h. This would complement the findings from the 48 h timepoint in the SK-GT-4 cells in which we observed that the ICB-FLOT combination decreased γH2AX expression (a surrogate marker of the induction of DNA damage response signalling^58^) suggesting that ICB may inhibit the induction of DNA damage signalling. Other studies which have demonstrated that PD-L1 signalling regulates the induction of the DNA damage response signalling in tumour cells^18,55,56^ which would support the hypothesis that is being presented with our data.

The expression of different IC proteins was differentially regulated by different pathways in the OE33 versus the SK-GT-4 cell line. STAT3 signalling had no effect on IC expression in the SK-GT-4 cell line and minimally affected the expression of only three ICs in the OE33 cell line (PD-1, LAG-3 and A2aR). STAT3 signalling mediates upregulation of PD-1 on the surface of T cells and subsequent STAT3 signalling blockade downregulates PD-1 expression^38^. Conversely, in this study STAT3 inhibition increased PD-1 expression on the surface of OE33 cells. These findings may be attributed to aberrant signalling in tumour cells compared with non-cancer cells, such as immune cells as studies have shown that PD-1 has opposing roles in T cell biology in comparison with tumour cell biology. PD-1 activation in T cells inhibits MAPK signalling attenuating pro-survival signalling in T cells^66^. However, PD-1 activates MAPK signalling in thyroid cancer cells promoting tumour cell survival^67^. Additionally, STAT3 signalling regulates PD-L1 expression on the surface of colon cancer cells^39^ however, STAT3 blockade had no significant effect on PD-L1 expression in OAC cells in this study. These discrepancies in PD-L1 regulation between these two tumour cell types may reflect the different signalling networks present and regulatory mechanisms in both tumour cell types which arise from cells types at opposite ends of the gastrointestinal tract from different cells of origin and completely different microenvironments.

However, MEK signalling had a more substantial effect in the regulation of a variety of ICs in both the OE33 and SK-GT-4 cell lines with differential effects observed between the two cell lines, upregulating certain ICs in one cell line while decreasing those ICs in the other cell line. Additionally, the MEK-mediated regulation of IC expression was altered by the addition of FLOT treatment, basally certain ICs were not affected by MEK inhibition however, in the presence of FLOT, inhibition of MEK signalling decreased or increased particular ICs. This may be attributed to FLOT-induced genotoxic stress and the activation of subsequent pro-survival signalling pathways such as RAS/RAF/MEK/ERK or IL-6/STAT3 which substantially alters a complex array of intracellular signalling networks, which may likely all interconnect in the regulation of ICs via positive or negative feedback loops. Additionally, the FLOT-induced upregulation of ICs may be a survival mechanism employed by these cells to help promote their survival under adverse conditions which could be mediated by MEK signalling. However, basally their expression may be regulated by different pathways and could explain why MEK signalling has no effect on the expression of particular ICs basally.

In summary, the findings from this study highlight the novel immune-independent functions of IC tumour cell-intrinsic signalling in OAC cells promoting a range of hallmarks of cancer including promoting tumour cell growth and proliferation, enhancement of a cancer stem-like phenotype and enhancement of DNA repair. Importantly, blockade of the PD-1 signalling axis suppressed tumour cell growth, decreased cancer stem-like marker ALDH and expression of DNA repair genes alone and in combination with the FLOT chemotherapy regimen. Combining PD-L1, PD-1 or A2aR ICB with the FLOT regimen synergistically enhanced chemotherapy cytotoxicity in OAC cells. This highlights a strong clinical rational for combining ICB with the first-line chemotherapy regimens to not only reinvigorate anti-tumour immunity and prevent immune exhaustion but to directly enhance the cytotoxicity of FLOT via inhibition of immune-independent hallmarks of cancer mediated by IC-intrinsic signalling in OAC cells.

## Supplementary Information

Supplementary Information.

## References

[1] Pera M, Manterola C, Vidal O, Grande L (2005). Epidemiology of esophageal adenocarcinoma. J. Surg. Oncol..

[2] Kubo A, Corley DA, Jensen CD, Kaur R (2010). Dietary factors and the risks of oesophageal adenocarcinoma and Barrett’s oesophagus. Nutr. Res. Rev..

[3] Villanueva L (2020). Total neoadjuvant chemotherapy with FLOT scheme in resectable adenocarcinoma of the gastro-oesophageal junction or gastric adenocarcinoma: Impact on pathological complete response and safety. Ecancermedicalscience.

[4] Huang F-L, Yu S-J (2018). Esophageal cancer: Risk factors, genetic association, and treatment. Asian J. Surg..

[5] Moehler, M. H. *et al.* CheckMate 649: A randomized, multicenter, open-label, phase III study of nivolumab (NIVO) + ipilimumab (IPI) or nivo + chemotherapy (CTX) versus CTX alone in patients with previously untreated advanced (Adv) gastric (G) or gastroesophageal junction (GEJ) . *J. Clin. Oncol.* **36**, TPS192–TPS192 (2018).

[6] Wang YJ, Fletcher R, Yu J, Zhang L (2018). Immunogenic effects of chemotherapy-induced tumor cell death. Genes Dis..

[7] Sun F, Cui L, Li T, Chen S, Song J, Li D (2019). Oxaliplatin induces immunogenic cells death and enhances therapeutic efficacy of checkpoint inhibitor in a model of murine lung carcinoma. J Recept. Signal Transduct. Res..

[8] Haruna M, Iwahori K, Kanazawa T, Yamamoto Y, Goto K, Kawashima A, Morimoto-Okazawa A, Funaki S, Shintani Y, Kumanogoh A, Wada H (2020). Docetaxel upregulates hmgb1 levels in non-small cell lung cancer. Biol. Pharm. Bull..

[9] Cottone A, Gualteroni C, Perrotta C, Bianchi ME, Rovere-Querini P, Manfredi A (2015). 5-Fluorouracil causes leukocytes attraction in the peritoneal cavity by activating autophagy and HMGB1 release in colon carcinoma cells. Int. J. Cancer.

[10] Galetto S, Forno S, Moro F, Mussa A, Matera L (2003). Drug- and cell-mediated antitumor cytotoxicities modulate cross-presentation of tumor antigens by myeloid dendritic cells. Anticancer Drugs.

[11] Galluzzi L, Buqué A, Kepp O, Zitvogel L, Kroemer G (2017). Immunogenic cell death in cancer and infectious disease. Nat. Rev. Immunol..

[12] Serrano-del Valle A, Anel A, Naval J, Marzo I (2019). Immunogenic cell death and immunotherapy of multiple myeloma. Front. Cell Dev. Biol..

[13] Kroemer G, Galluzzi L, Kepp O, Zitvogel L (2013). Immunogenic cell death in cancer therapy. Annu. Rev. Immunol..

[14] Hsu W, Hsu YH, Chan LC, Yu WH, Cha JH, Chen CT, Liao HW, Kuo CW, Khoo KH, Hsu JL, Li CW, Lim SO, Chang SS, Chen YC, Ren GX, Hung MC (2018). STT3-dependent PD-L1 accumulation on cancer stem cells promotes immune evasion. Nat. Commun..

[15] Shi Z, Miao J, Du S, Ai S, Xu E, Feng M, Song J, Guan W (2019). Adenosine interaction with adenosine receptor A2a promotes gastric cancer metastasis by enhancing PI3K-AKT-mTOR signaling. Mol. Biol. Cell.

[16] Cao Y (2013). Tim-3 expression in cervical cancer promotes tumor metastasis. PLoS ONE.

[17] Raniszewska A, Polubiec-Kownacka M, Rutkowska E, Domagala-Kulawik J (2019). PD-L1 expression on lung cancer stem cells in metastatic lymph nodes aspirates. Stem Cell Rev. Rep..

[18] Sato H (2017). DNA double-strand break repair pathway regulates PD-L1 expression in cancer cells. Nat. Commun..

[19] Zhong F, Cheng X, Sun S, Zhou J (2017). Transcriptional activation of PD-L1 by Sox2 contributes to the proliferation of hepatocellular carcinoma cells. Oncol. Rep..

[20] Gupta HB (2016). Tumor cell-intrinsic PD-L1 promotes tumor-initiating cell generation and functions in melanoma and ovarian cancer. Signal Transduct. Target. Ther..

[21] Longley DB, Harkin DP, Johnston PG (2003). 5-Fluorouracil: mechanisms of action and clinical strategies. Nat. Rev. Cancer.

[22] Martinez-Balibrea E (2015). Tumor-related molecular mechanisms of oxaliplatin resistance. Mol. Cancer Ther..

[23] Pienta KJ (2001). Preclinical mechanisms of action of docetaxel and docetaxel combinations in prostate cancer. Semin. Oncol..

[24] Neuzillet C (2014). MEK in cancer and cancer therapy. Pharmacol. Ther..

[25] Johnson DE, O’Keefe RA, Grandis JR (2018). Targeting the IL-6/JAK/STAT3 signalling axis in cancer. Nat. Rev. Clin. Oncol..

[26] Gong, X., Fan, L. & Wang, P. MEK inhibition by trametinib overcomes chemoresistance in preclinical nasopharyngeal carcinoma models. *Anticancer. Drugs* **32**, (2021). 10.1097/CAD.0000000000001092 34282746

[27] Kashyap T (2018). Crosstalk between Raf-MEK-ERK and PI3K-Akt-GSK3β signaling networks promotes chemoresistance, invasion/migration and stemness via expression of CD44 variants (v4 and v6) in oral cancer. Oral. Oncol..

[28] Jin W (2003). Roles of the PI-3K and MEK pathways in Ras-mediated chemoresistance in breast cancer cells. Br. J. Cancer.

[29] Stutvoet TS (2019). MAPK pathway activity plays a key role in PD-L1 expression of lung adenocarcinoma cells. J. Pathol..

[30] Timme S (2014). STAT3 expression, activity and functional consequences of STAT3 inhibition in esophageal squamous cell carcinomas and Barrett’s adenocarcinomas. Oncogene.

[31] O’ Sullivan, K. E. *et al.* pstat3 levels have divergent expression patterns and associations with survival in squamous cell carcinoma and adenocarcinoma of the Oesophagus. *Int. J. Mol. Sci.* **19**, (2018). 10.3390/ijms19061720 PMC6032321 29890775

[32] Lord RV, O’Grady R, Sheehan C, Field AF, Ward RL (2000). K-ras codon 12 mutations in Barrett’s oesophagus and adenocarcinomas of the oesophagus and oesophagogastric junction. J. Gastroenterol. Hepatol..

[33] Fichter CD (2011). Occurrence of multipolar mitoses and association with Aurora-A/-B kinases and p53 mutations in aneuploid esophageal carcinoma cells. BMC Cell Biol..

[34] Umstead M, Xiong J, Qi Q, Du Y, Fu H (2017). Aurora kinase A interacts with H-Ras and potentiates Ras-MAPK signaling. Oncotarget.

[35] Davern M (2021). Chemotherapy regimens induce inhibitory immune checkpoint protein expression on stem-like and senescent-like oesophageal adenocarcinoma cells. Transl. Oncol..

[36] Bozorgmehr N (2021). Expanded antigen-experienced CD160+CD8+effector T cells exhibit impaired effector functions in chronic lymphocytic leukemia. J. Immunother. Cancer.

[37] Tan, C. L. *et al.* CD160 Stimulates CD8&lt;sup&gt;+&lt;/sup&gt; T cell responses and is required for optimal protective immunity to &lt;em&gt;Listeria monocytogenes&lt;/em&gt; *ImmunoHorizons* **2**, 238 LP–250 (2018). 10.4049/immunohorizons.1800039 PMC7464592 31022694

[38] Zhang C (2018). Docetaxel down-regulates PD-1 expression via STAT3 in T lymphocytes. Clin. Lung Cancer.

[39] Xi X (2021). Interleukin-22 promotes PD-L1 expression via STAT3 in colon cancer cells. Oncol. Lett..

[40] Ozawa N (2021). PD-L1 upregulation is associated with activation of the DNA double-strand break repair pathway in patients with colitic cancer. Sci. Rep..

[41] Tu X (2019). PD-L1 (B7–H1) competes with the RNA exosome to regulate the DNA damage response and can be targeted to sensitize to radiation or chemotherapy. Mol. Cell.

[42] Lynam-Lennon N (2016). Low miR-187 expression promotes resistance to chemoradiation therapy in vitro and correlates with treatment failure in patients with esophageal adenocarcinoma. Mol. Med..

[43] Lynam-Lennon N (2010). Alterations in DNA repair efficiency are involved in the radioresistance of esophageal adenocarcinoma. Radiat. Res..

[44] Lynam-Lennon N (2012). MicroRNA-31 modulates tumour sensitivity to radiation in oesophageal adenocarcinoma. J. Mol. Med..

[45] Lynam-Lennon N (2017). MicroRNA-17 is downregulated in esophageal adenocarcinoma cancer stem-like cells and promotes a radioresistant phenotype. Oncotarget.

[46] Liu N (2016). Programmed death 1 induces cell chemoresistance to 5-fluorouracil in gastric cancer cell lines. Transl. Cancer Res..

[47] Sato A, Yasuhara T, Permata TBM, Hagiwara Y, Isono M, Nuryadi E, Sekine R, Oike T, Kakoti S, Yoshimoto Y, Held KD, Suzuki Y, Kono K, Miyagawa K, Nakano T, Shibata A (2017). DNA double-strand break repair pathway regulates PD-L1 expression in cancer cells. Nat. Commun..

[48] Permata Y, Sato H, Yasuhara T, Oike T, Gondhowiardjo S, Held KD, Nakano T, Shibata A (2019). Base excision repair regulates PD-L1 expression in cancer cells. Oncogene.

[49] Tu B, Zhang Y, Zhang C, Kahila M, Nowsheen S, Yin P, Yuan J, Pei H, Li H, Yu J, Song Z, Zhou Q, Zhao F, Liu J, Dong H, Mutter RW, Lou Z (2019). PD-L1 (B7–H1) competes with the RNA exosome to regulate the DNA damage response and can be targeted to sensitize to radiation or chemotherapy. Mol. Cell.

[50] Kim S (2019). Programmed cell death ligand-1-mediated enhancement of hexokinase 2 expression is inversely related to T-cell effector gene expression in non-small-cell lung cancer. J. Exp. Clin. Cancer Res..

[51] Zhang P, Su D-M, Liang M, Fu J (2008). Chemopreventive agents induce programmed death-1-ligand 1 (PD-L1) surface expression in breast cancer cells and promote PD-L1-mediated T cell apoptosis. Mol. Immunol..

[52] Degirmenci, U., Wang, M. & Hu, J. Targeting aberrant RAS/RAF/MEK/ERK signaling for cancer therapy. *Cells* **9**, (2020). 10.3390/cells9010198 PMC7017232 31941155

[53] Ebert PJR (2016). MAP kinase inhibition promotes T cell and anti-tumor activity in combination with PD-L1 checkpoint blockade. Immunity.

[54] Yan F (2016). Elevated cellular PD1/PD-L1 expression confers acquired resistance to cisplatin in small cell lung cancer cells. PLoS ONE.

[55] Permata TBM (2019). Base excision repair regulates PD-L1 expression in cancer cells. Oncogene.

[56] Sato H, Jeggo PA, Shibata A (2019). Regulation of programmed death-ligand 1 expression in response to DNA damage in cancer cells: Implications for precision medicine. Cancer Sci..

[57] Meng X, Yang S, Camp VJA (2020). The interplay between the DNA damage response, RNA processing and extracellular vesicles. Front. Oncol..

[58] Mah L-J, El-Osta A, Karagiannis TC (2010). γH2AX: A sensitive molecular marker of DNA damage and repair. Leukemia.

[59] Shi L (2019). Adenosine interaction with adenosine receptor A2a promotes gastric cancer metastasis by enhancing PI3K–AKT–mTOR signaling. Mol. Biol. Cell.

[60] Davern M (2021). The tumour immune microenvironment in oesophageal cancer. Br. J. Cancer.

[61] Kleffel S (2015). Melanoma cell-intrinsic PD-1 receptor functions promote tumor growth. Cell.

[62] Wang Q-E (2015). DNA damage responses in cancer stem cells: Implications for cancer therapeutic strategies. World J. Biol. Chem..

[63] Reya T, Morrison SJ, Clarke MF, Weissman IL (2001). Stem cells, cancer and cancer stem cells. Nature.

[64] Gao M (2019). Direct therapeutic targeting of immune checkpoint PD-1 in pancreatic cancer. Br. J. Cancer.

[65] Jiang J (2013). Decreased galectin-9 and increased Tim-3 expression are related to poor prognosis in gastric cancer. PLoS ONE.

[66] Sun C, Mezzadra R, Schumacher TN (2018). Regulation and function of the PD-L1 checkpoint. Immunity.

[67] Liotti F (2021). PD-1 blockade delays tumor growth by inhibiting an intrinsic SHP2/Ras/MAPK signalling in thyroid cancer cells. J. Exp. Clin. Cancer Res..

