# Supplementary material for: PD-1 blockade enhances chemotherapy toxicity in oesophageal adenocarcinoma
Source: Sci Rep. 2022 Feb 28;12:3259. doi: 10.1038/s41598-022-07228-x (PMC8885636; doi:10.1038/s41598-022-07228-x)
Supplement: Supplementary file 1 — Supplementary Information. [file 41598_2022_7228_MOESM1_ESM.docx]

Supplemental

**Figure S1: Effect of FLOT on IC co-expression profiles on the surface of OAC cells longitudinally *in vitro*.** OE33 cells were treated with vehicle control (veh) or FLOT for 48h (T1), washed twice and allowed to grow for an additional 48h (T2) after which the cells were sub-cultured in new flasks and left to recover for 3 weeks (T3). Following complete recovery, the cells were sub-cultured 1 in 2 and screened for IC expression 3 days later (T4). The frequency of PD-L1^+^PD-L2^+^ cells, PD-L1^+^PD-1^+^ cells, PD-L2^+^PD-1^+^ cells, LAG-3^+^CD160^+^ cells, LAG-3^+^A2aR^+^ cells, LAG-3^+^TIGIT^+^ cells, PD-L1^+^PD-L2^+^PD-1^+^ cells, LAG-3^+^TIGIT^+^A2aR^+^ cells, LAG-3^+^ A2aR^+^ TIGIT^+^ cells, LAG-3^+^CD160^+^TIGIT^+^ cells, LAG-3^+^A2aR^+^CD160^+^ cells and LAG-3^+^A2aR^+^CD160^+^TIGIT^+^ cells were assessed on the surface of OE33 cells longitudinally by flow cytometry at T1, T2, T3 and T4. Experiments repeated for n=4 independent experimental repeats with singlet technical replicates. Two-way Anova using Benjamini and Hochberg to correct for false discovery rate *p<0.05, **p<0.01.

**Figure S2: Inhibition of STAT3 signalling exclusively regulates IC expression in OE33 cells and not SK-GT-4 cells.** OE33 cells and SK-GT-4 cells were treated with and without FLOT chemotherapy regimen in the absence and presence of a STAT3 inhibitor (STAT3i) for 48h and the expression of IC ligands (PD-L1, PD-L2) and IC receptors (PD-1, TIGIT, LAG-3 and A2aR) on the surface of OAC cells was determined by flow cytometry. *p<0.05 and **p<0.01, experiments repeated for n=3 independent experimental repeats with singlet technical replicates, Kruskall-Wallis.

**Figure S3: Effect of PD-1, PD-L1 and A2aR blockade alone and in combination with FLOT on the total cell counts of viable, early stage apoptotic, necrotic and late stage apoptotic/dead cells.** OE33 cells and SK-GT-4 cells were treated with nivolumab (10 μg/ml), atezolizumab (10 μg/ml) or A2aR antagonist (3 μM) in the absence or presence of FLOT regimen for 48h. The total cell counts of necrotic cells ((A) AV^-^PI^+^), late stage apoptotic/dead cells ((B) AV^+^PI^+^), viable cells ((C) AV^-^PI^-^) and early stage apoptotic cells ((D) AV^+^PI^-^) was determined by flow cytometry using annexin V propidium iodide assay. Experiments repeated for n=4 independent experimental repeats with singlet technical replicates. *p<0.05, **p<0.01 and ***p<0.001, Kruskal-Wallis.
